# Macroscopic Markers of Dolphin Healing at Sea Linked to Immunity

**DOI:** 10.3390/ani16020305

**Published:** 2026-01-19

**Authors:** Ann Weaver

**Affiliations:** 1 Good-Natured Statistics Consulting, Lake City, FL 32024, USA; annstats54@gmail.com; 2 Maples Center for Forensic Medicine, College of Medicine, University of Florida, Gainesville, FL 32608, USA

**Keywords:** free-ranging dolphins, bottlenose dolphins, wound healing, immunity, unaided healing, pigment patterns, shark bites, dolphin dermatitis inflammation, proliferation, remodeling, atrophic scars, hyperpigmentation, cetaceans

## Abstract

Wound healing has been studied extensively in humans and lab animals, but not in dolphins. Severe human wounds require extensive medical intervention to avoid infection. Yet severe wounds on free-ranging dolphins heal without infection in microbial-infested seas, a compelling distinction. An eye-witnessed shark attack on a yearling bottlenose dolphin yielded 8 years of macroscopic markers on a live recuperating dolphin by known days of healing. In total, 106 healing histories were generated from the author’s 20-year ethological study of free-ranging bottlenose dolphins (*Tursiops truncatus*) in St. Petersburg, FL, USA. Results show that unaided wound healing at sea involves two consecutive macroscopic pigment patterns, wounds form preliminary seals by 4–8 weeks, and most heal to atrophic scars that remodel for years. Macroscopic markers in live recuperating dolphins show strong matches with macroscopic wound patterns in stranded Fraser’s dolphins (*Lagenodelphis hosei*), demonstrating links between macroscopic markers and immune activities. This is the first study to link macroscopic markers visible as healing-related pigment patterns to immunity. Macroscopic markers are conservation tools for tracking anthropogenic impacts on increased susceptibility to infection at sea and could lead to novel therapies in veterinary and human regenerative medicine.

## Simple Summary

Accurate knowledge of healing benefits everyone. A pressing human need is learning how to heal without infection. Animal medical models of healing often lead to novel therapies, so new animal models are always sought. This study presents a new medical model: dolphins wounded at sea heal without medical intervention and commonly without infection despite constant exposure to microbial-rich seas. This daunting medical mystery has been illuminated in a set of 106 detailed longitudinal healing histories and scar searches, which illustrate several macroscopic markers of healing visible to boat-based observers. Further, strong matches with macroscopic markers of the most histologically detailed study of free-ranging dolphin healing to date establish links between markers and underlying immune phases of inflammation, proliferation, and remodeling. These results are pertinent to people interested in the healing process, cosmetic outcomes of scarring, and dolphin stranding, natural history, and conservation studies.

## 1. Introduction

The treatment of large, infected soft-tissue wounds remains a medical challenge [1,2,3,4]. In humans, large wounds demand medical interventions of hemostasis to stop bleeding, antisepsis to control infection so the patient can survive long enough to heal, and surgical attempts to limit scar formation, yet infection and heavy scarring are often unavoidable [4]. Likewise, lack of intervention leads to serious human complications [4]. Even with medical intervention, humans with shark bites that expose them to marine-borne bacteria (e.g., *Vibrio*) develop infections that resist standard antibiotics [4], and a human with a full-thickness “core sample” cookie-cutter shark wound required more than six months to heal despite a skin graft [5]. In contrast, a cookie-cutter shark wound on a Fraser’s dolphin filled with granulation tissue within days and completely closed in the next two months without infection [6]. Live free-ranging dolphins heal minor to major wounds without medical intervention and commonly without developing infections [7,8,9,10,11] or chronic (unhealing) wounds despite constant exposure to microbial-rich seas [12]. These compelling distinctions make the unaided healing of free-ranging dolphins a vital area of inquiry. They have veterinary implications for triage decisions and the advisability of interventions for dolphins suffering natural and anthropogenic injuries [13]. They have medical implications for healing and scarring that occur without the crucial immunological contributions of hair follicles and glands, as in terrestrial mammals and haired marine mammals (polar bears, otters, pinnipeds). The purpose of the study is to highlight the macroscopic markers of dolphin wound healing at sea and show their connection to immune phases.

Human cutaneous wounds scar differently from dolphins. In humans, the original skin architecture is wholly ablated—damaged nerves and skin appendages (hairs, sweat glands, and sebaceous glands) are not replaced in scar tissue [14]—but in dolphins, epidermis is replaced fully and rapidly (reepithelialization) [6,15]. In humans, skin conditions like acne and chickenpox often leave pitted or atrophic scars [16], but in dolphins, even the most serious skin conditions heal scar-free ([12] Vol. 14, Dermatitis).

Cetacean healing is a topic about which little is known but much has been speculated, and is rife with superlatives about its speed and completeness. Early claims about remarkably fast proliferation rates of dolphin skin [17], rapid healing compared to terrestrial mammals [18], and other potential roles of specialized cetacean skin in healing [1] prompted Bruce-Allen and Geraci [15] to test them with a minor wounding study. Initial conclusions that dolphin healing was not qualitatively different from that of lab animals and humans were soon overturned by evidence of rapid proliferation and turnover [19]. Most authors since have characterized dolphin healing as superlative [1,8,9,10,13,20,21,22,23], despite limited data from sighting gaps and small sample sizes.

**Figure 1:**
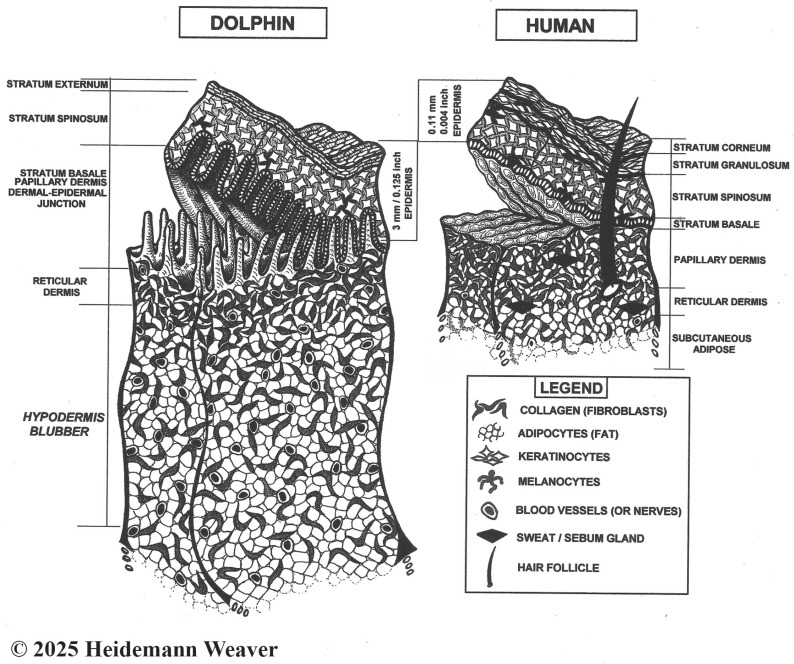
Not-to-scale stylized images of bottlenose dolphin skin specializations following [103] and human skin. Most human skin is paper-thin [104] with four epidermal layers (palms and soles have five layers). Dolphin skin is 20 times [45] to 50 times [27] thicker than human skin. It further departs from the typical mammalian design, being smooth, taut, and hairless, with three epidermal layers, extreme thickness, a lack of hair follicles and glands, and a heavily convoluted dermal–epidermal junction. Comparatively, dermal–epidermal junction convolutions are shallow in pigs and humans and smooth in hairy lab animals [97]. Due to layering and thickness differences, the 4 mm wound, often used in lab animal healing studies, creates a full-thickness wound down to muscle; in dolphins, the same depth creates a superficial partial-thickness wound that barely penetrates the dermis. For comparison, bull shark (*Carcharhinus leucas*) teeth average 13–38 mm long, 3–9 times the depth of the popular 4 mm excision. Due to dolphin epidermal thickness, an apparently serious wound may involve only superficial epidermal damage without more serious damage. Yet apparently superficial shark tooth rakes inside a crescent bite that do not break the skin can remain visible for a decade ([12], Vol. 4 Dorsum Bites, Ch. 2 Keynote Case, Bet). The organization of the epidermis of mice, rats, and rabbits is like humans but too thin and hairy for reepithelialization studies. In contrast, hairless pig and human skin is thick enough for repeated partial wounding studies [97].

Evidence was needed from longitudinal healing studies. The few data on dolphin healing at sea without medical intervention are mainly from bottlenose dolphins, *Tursiops truncatus*, ecologically generic [24] but otherwise socially complex [25] and physically specialized. Their biopsychosocial exaggerations of terrestrial mammal characteristics are due to an array of evolutionary novelties [26] associated in many cases with gene loss [27]. Social complexities include the rare fission–fusion social organization, high intelligence [28,29,30,31], imitation and pointing [32], cooperation [33], self-identifying signature whistles [34], mirror self-recognition [35], self-decoration [25], the impressive communicative repertoire of aerial behaviors [36], promiscuous sexuality, maternal behaviors [37] and male alliances [38]. Physical adaptations include streamlining, loss of hair and glands, 80–90% exchange of lung air per breath [39] (humans exchange 15–20% [40]), and rare unihemispheric slow wave sleep [41]. Dolphin skin (Figure 1) reflects a range of morphological, physiological, and overall anatomical innovations [42,43,44,45,46,47,48]. Massive vascular plexuses called retia mirabilia support their complex physiological diving reflex [49,50,51,52].

### 1.1. Eyewitness to Shark–Dolphin Incident Led to the Faithful Canvas Collection

Free-ranging dolphins who survive traumatic wounding at sea, whether from requiem shark bites or propeller strikes, heal without medical intervention and typically without being observed. On 10 June 2016, we had been studying five free-ranging resident bottlenose dolphins from a small boat (Figure 2) for many minutes when we witnessed a shark attack the yearling male. This striking incident took only moments, culminating in 11 bites, and enabled us to document the reactions of the bitten yearling Saga, his mother, and his social group before, during, and after the incident, as well as those of the five nearby dolphins who joined the wholesale flight from the scene [53]. Moreover, over the next 8 years (2016–2024), this well-habituated mother–calf pair allowed us to systematically track and photo-document the calf Saga’s macroscopic healing patterns, based on known days of healing. I tracked his healing by scrutinizing my photo archives and selecting suitably informative photos; the resulting account of Saga’s recovery and remodeling was my first healing history (excerpts in Figure 3, Figure 4, Figure 5, Figure 6, Figure 7, Figure 8, Figure 9, Figure 10, Figure 11, Figure 12, Figure 13, Figure 14, Figure 15, Figure 16, Figure 17, Figure 18, Figure 19, Figure 20, Figure 21, Figure 22, Figure 23, Figure 24, Figure 25, Figure 26, Figure 27, Figure 28, Figure 29, Figure 30, Figure 31, Figure 32, Figure 33, Figure 34, Figure 35, Figure 36, Figure 37, Figure 38, Figure 39, Figure 40 and Figure 41).

This led me to write another 105 healing histories and scar searches on free-ranging bottlenose dolphins in my study area (*N* = 474 identified dolphins, 45% residents, Figure 3, Figure 4, Figure 5, Figure 6, Figure 7, Figure 8, Figure 9, Figure 10, Figure 11, Figure 12, Figure 13, Figure 14, Figure 15, Figure 16, Figure 17, Figure 18, Figure 19, Figure 20, Figure 21, Figure 22, Figure 23, Figure 24, Figure 25, Figure 26, Figure 27, Figure 28, Figure 29, Figure 30, Figure 31, Figure 32, Figure 33, Figure 34, Figure 35, Figure 36, Figure 37, Figure 38, Figure 39, Figure 40 and Figure 41). They are organized into 14 types of wounds, dermatitises, and organic disorders (body bites, head bites, dorsum bites, dorsal fin wounds, peduncle bites, propeller strikes, curvilinear tooth rakes, paralysis, seizures, and dermatitises) and show the range of wound types and survivable severity levels. The compilation is the multi-volume set, *Dolphin Healing at Sea without Medical Intervention: The Art and Science of Skin as the Faithful Canvas* [12]. Each healing history begins with its contributions to our knowledge of dolphin healing at sea without medical intervention, followed by chronological descriptions and photos of macroscopic markers (2500 pp). Photo legends list the days of healing since the wound occurred, the date the dermatitis developed, or the first date we saw the condition; the dolphin’s age and gender when known; the photo date; water temperature; and days since the previous photo. Few studies have examined the healing or long-term stability of scars in free-ranging cetaceans, and no standardized technique exists for quantifying wound scar characteristics [54]. Thus, as I wrote the healing histories, I simultaneously developed a standardized photo-illustrated glossary ([12] Vol. 1, Ch. 2, available on request: annstats54@gmail.com). Standardized terminology provides a robust basis for repeatable and consistent analyses during healing studies.

The healing histories led to 36 discoveries, several of which are highlighted in this study. Foundational discoveries are that dolphin skin heals bruises and dermatitises without scarring yet accurately portrays every detail of wounds from minor slices to major shark bites as permanent scars. Dolphin skin is thus a canvas that records virtually every strike against it faithfully. Hereafter, for brevity, the healing histories of live recuperating dolphins are collectively referred to as the “Faithful Canvas collection” or [12].

### 1.2. Publications on Dolphin Wounding and Scarring at Sea

Early studies of dolphin wound healing at sea were hampered by wounds of unknown age, low survey effort, gaps in sightings (infrequent resights), poor photographic detail, small sample sizes, and a lack of a standardized vocabulary. For example, Corkeron et al. [9] observed 21 dolphins with fresh wounds but could estimate healing rates for only three due to infrequent resights of the other 18. Caldwell et al.’s [55] description of a shark bite on a bottlenose dolphin as “fresh but nearly healed” (p. 8) suffered from the lack of standardized vocabulary. Most early reports measured shark marks on dolphins as indirect indications of predatory pressure; few shark–dolphin interactions were observed directly [9,11,56,57,58,59,60,61,62,63,64,65,66,67,68,69,70,71,72,73,74,75,76,77,78,79,80,81]. Shark bite wounds and scars are defined as typically crescent-shaped, jagged, and consisting of widely spaced tooth marks and rakes not attributable to other dolphins [66,74,82]. The Faithful Canvas [12] defines three parts of a crescent: cranial leg (straight portion of the U-shaped crescent towards the dolphin’s head or cranial aspect), caudal leg (straight portion of the U-shaped crescent towards the dolphin’s flukes or caudal aspect), and apex (rounded junction where cranial and caudal legs meet, shark jaw symphysis). Fewer early healing data are available on vessel and propeller strikes [10,83,84,85,86,87,88].

Lockyer and Morris [7,8,89] were among the first to report the value of systematic observations of body scars for informing a species’ socioecology. Following their lead [89], Olaya-Ponzone and colleagues [21] classified five levels of wound type, severity, and survival chances by photographically tracking fresh lacerations before, during, and after wounding on five common dolphins (*Delphinus delphis*) off the southern Iberian Peninsula, an area of intense anthropogenic activity (Table 1). Based on sighting gaps, maximum possible healing times ranged from 1 to 6.3 months, which corresponded to their wound severity schema. Luksenburg [90] defined 11 injury categories linked to either human-related activities or natural causes, but resights were infrequent, so healing times of fresher wounds were not estimated. The Marine Mammal Protection Act (MMPA) requires the National Oceanic and Atmospheric Administration’s (NOAA) National Marine Fisheries Service (NMFS) permit holders to broadly distinguish between serious and non-serious injuries to marine mammals, defining a “serious injury” as an injury with a greater than 50% chance of leading to the animal’s death [91].

Two studies of dolphins provided macroscopic markers of healing days from propeller wounds of known age. Bloom and Jager [10] provided healing details of 11 serious lacerations after an old, lone bottlenose dolphin with full-body dermatitis was slashed on the right side of the dorsum by a boat engine propeller at dusk in late September. Healing was helped by a steady supply of salmon but hindered by cold water (10–14 °C, 50–57 °F), old age, poor physical condition, and sewage pollution. When struck, the dolphin immediately showed significant agitation. All his wounds were full-thickness; seven lesser lacerations penetrated the blubber (slashes 1, 6–11) and four severe lacerations penetrated muscle (slashes 2–5). His wounds bled for two days. Swollen wound edges gave a corrugated appearance that lasted past day 100. By day 3, sand and dirt lodged in the gaping wounds gave them a brown, grainy appearance. By day 8, the lesser lacerations were white and partly closed, almost closed by day 18, and healed by day 37; the scars remained hypopigmented, conspicuous, and swollen. By day 8, the four deepest lacerations still gaped, had pronounced swelling and inflammation (not described), and by day 12 had developed the buffering layer of dead and dying cells that functions as a scab [15]. By day 65, the deeper lacerations continued to close from the inside, and a thin hypopigmented line marked the wound bed. After 12 weeks/3 months, they healed into hypopigmented patches edged by “darker irregular areas like tendrils” (p. 63) that extended inward from the white scar toward its center, indicating early repigmentation. Bloom and Jager were the only authors to report infection, which they described as discolored, purulent tissue in slash 4, which by day 32 had expanded to a hand-sized, hypopigmented patch of textured, abscessed tissue. By day 128, or 4.3 months (p. 61), the “abscess scar site remained strikingly white [with an] irregular gray margin,” and laceration scars were highlighted by “a pale curved scar line in a recessed surrounding area of much darker pigmentation, especially on the more serious wounds.” In standardized Faithful Canvas terms, this is an atrophic scar in the bi-colored dark-to-light pigment pattern. Humans with matrix-deposition problems due to diet or disease develop weak scars whose strength is greatly compromised [92]. Whether dolphins in poor condition do as well remains to be determined.

Elwen and Leeney [84] provided healing details of nine full-thickness lacerations after a Heaviside’s dolphin (*Cephalorhynchus heavisidii*) was struck by a boat engine propeller on the left side of the peduncle. The caudal half of the wounds was more severe. The injured animal (sex unknown) was observed 11 times and photographed 10 times between 13 June 2008 and 4 August 2010. Wounds were obtained in February 2010. Although time since the previous sighting was excluded (i.e., the oldest possible wound age could not be independently estimated), descriptions of pale pink muscle with a clear differentiation between skin, blubber, and muscle without blood suggested that the wounds were fresh. By an estimated 19–23 days post injury, skin covering the lesser cuts created a pale center and dark outline. By day 39 (final sighting of the summer field season, 22 March 2010), the lesser anterior scars showed near-complete healing with the wounds only visible as a darker outline and paler center discoloration with no obvious indentation or swelling. Five months after the first observation of the wounds (4 August 2010, 174 days post injury), the authors of [84] described all injuries as healed and completely repigmented (no white scar tissue), but with clearly visible scars protruding slightly from the body. However, Figure 1H in [84] does not suggest protruding scar tissue although there is a clear bi-colored light-to-dark pigment pattern with some suggestions of scar atrophism. Wound depth was estimated visually.

### 1.3. Estimated Days to Healing on Wounded Dolphins at Sea

Dolphins with open wounds at sea are subject to microbial invasion until the wound has closed, so even broadly estimated days to a preliminary seal are informative about the potential duration of inflammation in dolphins at sea. The literature contains some estimates of healing times, but the major constraint is that researchers rarely know the age of the wounds they encounter at sea, which accounts for the tremendous variability in these estimates. Table 1 lists published estimates of healing days in ascending order and shows that estimates ranged from 23 to 584 days for various wound types and severities. Within wound types, estimated days to healing also ranged substantially: tooth rakes, 23–584 days [7,93,94,95]; propeller strikes, 39–100+ days [10,84]; shark bites, 28–180+ days; biopsies, 23–42 days [20,96]; and wounds from nebulous sources, 45–90 days [8,21]. Part of the variability emanates from differences in wound types (imposed or naturally occurring).

**Table 1:** Estimated days of dolphin wound healing at sea.

Source	Estimated Days to Sealing	Species, Wound Type, Comments
[96]	23	Bottlenose dolphins (*Tursiops truncatus*). Biopsy wounds. Healed in approximately 23 days.
[9]	28	Bottlenose dolphins. Shark bites. The worst bite was seen on dolphin B41, at most 17 days old, which had “healed substantially” (p. 8) by 28 days old at most. Photos and text lacked details; the wounded dolphin in Figure 1a, [9] had a degloved cranial leg, but the substantially healed dolphin in Figure 1b [9] appeared to have a degloved caudal leg. The wound was fully healed by August, 8 months later.
[11]	30	Bottlenose dolphin. Shark bite on the flank at least 3 cm deep. Estimated to close completely within 30 days/1 month and heal to a scar within 45 days/1.5 months.
[84]	39	Heaviside’s dolphin (*Cephalorhynchus heavisidii*). Nine full-thickness wounds from a boat engine propeller strike.
[20]	40–42	Bottlenose dolphins. Biopsy wounds.
[21]	45–60+	Common dolphins (*Delphinus delphis*). Level 2: Superficial dorsal fin lacerations, 49 days to seal. Level 3: Melon gouge, 60 days to seal. Levels 1–3 took 1–2 months to heal and probably correspond to superficial partial-thickness, deep partial-thickness, and shallow full-thickness wounds, respectively.
[8]	90	Bottlenose dolphin. A severe wound to the top of the head from the outboard engine skeg. Estimated to heal to a white scar within 90 days/3 months.
[10]	100+	Bottlenose dolphin. Eleven full-thickness boat engine propeller wounds. Still open after an estimated 65 days/2.2 months, and complete healing took 100+ days/3.3 months.
[21]	120–180+	Common dolphins. Level 4: Deep shark bite. Level 4: Major slash. Levels 4 and 5 were deeper wounds that penetrated the muscles and bled profusely (level 4) or reached the bone with significant tissue loss (level 5). The two dolphins with level 4 injuries (deep shark bite, major slash) took a maximum of 4 months and 6.3 months to heal, respectively. The dolphin with the worst (level 5) injury was only seen once.
[94]	150–365	Bottlenose dolphins. Tooth rakes. Several studies estimated that conspecific tooth rakes on free-ranging take 5–12 months to heal.
[95]	584	Bottlenose dolphins. Tooth rakes. Black parallel streaks indicate new tooth rakes. Shark Bay tooth rakes average 1.6 years to heal. Males with heavier tooth rake loads. Females healed tooth rakes faster.

### 1.4. Estimated Days to Healing in Humans and Lab Animals

In humans and lab animals, estimates of the number of days in each healing stage are based on wounds of known age, but also vary. For normal experimental incisions in animal studies, inflammation lasts the first 4–6 days; proliferation lasts from 4 to 14 days, provisional matrix synthesis peaks around 7–14 days and stimulates fibroblasts to begin synthesizing collagen I; net collagen synthesis continues for at least 4–5 weeks after wounding; and remodeling lasts 8–365 days [92]. In lab animals, inflammation lasts 1–10 days, proliferation lasts 1–90+ days, and remodeling lasts 1–300+ days [97], or inflammation lasts up to two weeks, and remodeling starts by three weeks and lasts a year [14].

### 1.5. Human and Dolphin Skin

As ancestral cetaceans evolved into obligate aquatic species, they co-opted unaided mammalian healing processes while evolving skin specially adapted to the chemical, hydrological, and microbial features of seawater that enabled life to originate on Earth. Sea water helps healing by supplying saline [54], moisture [98,99], and optimal pH = 8.1 for the proliferation of two major cell types in wound healing, keratinocytes and fibroblasts (pH = 7.2–8.3 [100]). Comparative genomics show that contemporary dolphins co-opted the accelerated skin production rates of wounded ancestors as their non-wounded status quo production rate yet retained the mammalian trait of accelerated epidermal production upon wounding. This accounted for the evolution of the extreme thickness, accelerated skin cell production, and rapid turnover [27,43,44,45]. Dolphin red blood cells, platelets, alpha granules [46] and adipose cells [47] are all significantly larger.

Skin is the largest organ, the only organ exposed to the outside environment, and first defense against pathogens [101]. It has complex mechanisms to protect itself and restore damaged tissues without septicemia [2]. As ancestral cetaceans evolved into a fully aquatic lifestyle, archetypal mammalian skin was drastically remodeled through streamlining, diving, and continuous swimming [42]. The cetacean epidermis and dermis provide the crucial boundary between the individual whale or dolphin and its environment [102], but, located outside the insulating blubber layer, this boundary is maximally exposed to physical, chemical, and biological stressors, including temperature extremes, pollution, and wounding [44].

The many singularities of cetacean skin may have potential significance for healing. Cetacean skin is mainly unique for its extreme thickness, high cellular turnover rate, and absence of terrestrial mammalian skin appendages [27]. Streamlining forces produce a drag-reducing skin [27,105,106] that is remarkably smooth compared to that of marine swimmers such as marlin, tuna, and pelagic sharks [107]. It is always wet, adapted to an obligate aquatic existence from torrid tropics to frigid poles, coastal to pelagic, and salty seas to estuaries to freshwater habitats. Correspondingly, it lacks sebaceous and sweat glands. It is naked. Cetacean skin differs significantly from terrestrial mammals by lacking hair follicles, though whisker vibrissae develop in utero and are visible on neonatal odontocetes [37]. It is tight. In contrast to loose-skinned lab animals like rodents and rabbits, dolphins are tight-skinned like humans and pigs. Finally, it is extraordinarily thick. Estimates of epidermal thickness range from 20 times [45] to 50 times thicker [27] than that of terrestrial mammals. Some unique features of cetacean skin likely serve healing [6,44]. Yet, despite the substantial literature on dolphin skin morphology, the intriguing potential of its unique anatomical specializations, histology, and fine structural features for cetacean healing without medical intervention remains understudied [102].

Mammalian skin is composed of three layers: outer epidermis, middle dermis, and lower hypodermis [108]. The epidermis is the outer layer of skin that creates a protective, waterproof barrier, whose color is determined by melanin concentrations. Paper-thin human skin [104] varies from 0.07 to 0.15 mm thick [109,110]. Human epidermis has five layers or strata (outside in: stratum corneum, lucidum, granulosum, spinosum, and basale; the stratum lucidum occurs only on palms and soles, so most human skin has four layers [97]). It is composed of 90% keratinocytes interspersed with melanocytes. In contrast, bottlenose dolphin epidermis has only three strata (outside in: stratum externum, spinosom, and basale) but is 2–4 mm thick [15], about the thickness of two American 25¢ coins (quarters), which 10–20 times thicker than terrestrial mammals and 30+ times thicker than in humans (Figure 1). Its thickness varies by body location, e.g., thicker on leading than trailing edges of dorsal fins [111] and flukes [112]. Epidermis is unvascularized [113]. In cetaceans, the lack of a distinct stratum granulosum (Figure 1) is a clear departure from terrestrial mammals and a major feature of the cetacean epidermis; its absence means that the stratum externum seamlessly transforms into the stratum spinosum [44]. Adaptations to a fully aquatic life also included modifications in epidermal gene expression, keratins, and lipids [44].

The outside layer of the three-layered epidermis (Figure 1) is the stratum externum (stratum corneum in terrestrial mammals [44]). In dolphins, a key aquatic adaptation is that the outer layers of flattened keratinocytes are incompletely cornified or keratinized [114] but see [39]. Keratinization is the process whereby living keratinocytes differentiate into the dead, hardened cells of the stratum corneum. Keratinocytes produce keratin, the tough, protective protein that makes up most of the structure of skin, hair, fur, nails, and horns. In terrestrial mammals, the outer skin is highly keratinized (cornified), which creates a compact, anucleated (dead) barrier against dehydration and physical injury [115]. In dolphins, keratinization is incomplete because the externum retains some features of living cells, such as nuclei (parakeratosis). Another key adaptation is its lipid component [112]. Cetacean skin includes a cell type not normally found in terrestrial mammals, lipokeratinocytes, a designation based on distinct keratinization patterns and very high levels of cellular lipogenesis that provide physical barriers against the hypertonic environment as well as buoyancy, streamlining, and insulation [44]. Dolphin lipokeratinocytes are 3–5 times more voluminous than human keratinocytes, with variability between and within cetacean species [116].

The middle and thickest epidermal layer is the stratum spinosum (Figure 1) or “spiny layer” composed of flat daughter keratinocyte cells held together with spiny projections [92] that form a permeability barrier [19]. Its dendritic cells fight infection [92], and its Langerhans cells bind antigens that invade damaged skin, alerting the immune system to their presence; cetacean Langerhans cells require further study [102]. The spinosum also contains large lipid droplets and laminar bodies, possibly enriched with antimicrobial peptides that protect against biofouling (the settling of larvae of sessile organisms, such as barnacles, on the skin [106]). However, little is known about the role of antimicrobial peptides in dolphin healing.

The bottom epidermal layer is the stratum basale (or germinativum). This is the site of the accelerated dolphin skin production. Basale cells form a single layer of keratinocytes and melanocytes, separated from the underlying dermis by a thin basement membrane, that line the entire heavily convoluted dermal–epidermal junction (Figure 1). Dermal–epidermal convolutions are generally true of mammals. However, rete prominence varies considerably; e.g., it is smooth in loose-skinned mice, rats, and rabbits [117]. In contrast, the highly folded architecture of the dolphin dermal–epidermal junction is another extraordinary characteristic of cetacean skin [44]. Histologically, these extensive folds vastly increase the surface area available for epidermal cell proliferation, which accounts for the basale layer’s exponential mitotic, proliferative, and sloughing capacities. Dolphin skin cells do not reproduce faster than human skin cells; there are 29 times more of them, and mitosis is continuous [118]. The thickness of bottlenose dolphin skin results from an 11.3 times greater production capacity [45] and 1.5 times longer cell lifespan [103] than in humans. In humans, epidermal replacement (reepithelialization [14]) occurs in the stratum granulosum, where each superficial cell overlies 25–30 germinal cells [15]. In bottlenose dolphins, reepithelialization starts in the dermal–epidermal junction, where each superficial cell overlies 870–876 germinal cells [103]; however, Menon et al.’s [44] cetacean skin review suggested that cell turnover rates warrant re-evaluation. Dolphin skin cells produced by the basale layer take 3–10 weeks to reach the surface, which is quick given the epidermal thickness [103]. The constant mitotic activity of the bottlenose dolphin’s basale cells replenishes the stratum externum every two hours, or 12 times a day. Sloughing of the dolphin stratum externum is 8–9 times faster than the human epidermal sloughing rate [19], which, at sea, maintains a smooth surface and acts as an antimicrobial frontline, limiting microbial colonization (biofouling [44]). Potential roles of cetacean skin microbial communities in immune-related functions are under investigation [101,106,119,120,121].

Keratinocytes of the stratum basale are surrounded by clear melanocytes that give rise to melanin, a pigment that accounts for skin color. In cetaceans, small grains of melanin (<5 μm) are prominent in gray skin, and larger grains are prominent in black skin [15]. Melanocytes produce melanin in response to ultraviolet (UV) sun exposure. Melanin covers keratinocyte DNA, protecting it from UV radiation [122]. In cetaceans, fully melanized epidermis is common [123]. However, in terrestrial mammals such as humans, cattle, and dogs, melanin does not extend beyond the stratum spinosum [123].

The second or middle skin layer, dermis, lies between the epidermis and hypodermis (Figure 1). It is a complex network of collagen, connective tissue, blood vessels, and nerve fibers (thermoreceptors for heat, mechanoreceptors for touch) with some adipocytes, elastic fibers, and extracellular matrix (ECM) for cushioning [6,23,44]. Dermal nerves and blood vessels supply the very thick epidermal layer; the latter augment crucial repair and replacement of blood vessels damaged by wounding (angiogenesis). Dolphin dermis has two layers. The upper layer is the papillary dermis, the structurally complex portion whose erect dermal papillae penetrate the lower epidermis and create the dermal–epidermal junction (Figure 1). Dermal papillae are evenly spaced, well-innervated, and well-vascularized. The deep dermal–epidermal interdigitations anchor the epidermis to the dermis robustly [44]. Papillary heights vary across the body, taller in the relatively thicker epidermis on fin leading edges than dorsal skin [112].

Under the dermal papillae zone, the lower reticular dermis is thicker and composed of dense connective tissue and high levels of collagen [6]. The dolphin reticular dermis grades into the blubber [124].

Further key dolphin dermal adaptations include the absence of hair follicles, sweat glands, and sebaceous glands, collectively known as skin appendages in terrestrial mammals [39,44]. All three skin appendages occur in the 0.5–3.0 mm thick human dermis [97]. Skin appendages directly contribute to healing by providing progenitor (stem) cells, glands with antimicrobial properties, and hair follicles, which produce granulation tissue that converges to fill open wounds [97]. The absence of skin appendages in cetaceans indicates where dolphin healing departed from the terrestrial mammal healing model by developing natural functional alternatives that are yet to be determined.

### 1.6. Blubber

The lowest layer is the hypodermis. Human hypodermis or subcutaneous tissue thickness varies significantly by body location, from less than 1 mm on the eyelids to over 2.5 cm on the abdomen and buttocks [110]. In cetaceans, it is the thick, specialized hypodermis called blubber [47]. Rich in adipocytes and connective tissue [6], blubber is another unique cetacean adaptation [124]. Its many functions include streamlining, thermoregulation, buoyancy, and energy storage [125] but see [126]. It is a highly organized biocomposite of connective tissue and adipocytes surrounded by a regular, three-dimensional weave of collagen and elastin structural fibers [127]. Blubber differs from adipose tissue by being thicker and more densely vascularized [128]. Whereas most types of adipose tissue contain some collagen, blubber is also distinct for the bulk of its collagen cargo and elastic fibers [128]. Collagen gives blubber its firm, tough, fibrous character, which drives its mechanical and functional properties. Blubber is further distinct for its dense vascularization [128] and specialized shunts that allow larger and swifter blood flow than is possible through capillaries alone [47].

Together, the dolphin’s dermis and blubber form a collagenous fortress that envelops the musculature, skeleton, and internal organs. Blubber overlies the underlying musculature almost continuously but is not tightly fixed to it [128]. Blubber thickness varies an average of 12–30 mm, accounting for 80–90% of the integument thickness in some odontocetes [47] but is either absent [128] or very thin [112] on fins and flukes. Blubber thickness generally tapers along the dolphin’s long axis, thinnest on the head and distal peduncle, thickest on the body under the dorsal fin (umbilical girth), and thicker in the dorsal than ventral and lateral regions [126]. On bottlenose dolphins, blubber is the thickest at the dorsum directly cranial to the dorsal fin and thinnest along the mid-lateral plane [129].

Potential healing roles for blubber’s collagen load, dense vascularization, and adipocyte stem cells remain to be explored, although several characteristics have possible implications. Like other skin layers, blubber is heterogeneous through its depth [47]. Its three blubber layers, created by thermal, structural, and chemical characteristics [128], are easily differentiated visually, histologically, biochemically, and by ultrasound [125,130]. The upper layer has a higher concentration of isovaleric acid [131], whose potential contribution to healing [1] warrants further study. The lower layer has a higher quantity of blood vessels in its upper portion [6,132]. During nutritional stress, lipid energy is mobilized inconsistently [125]: Melon lipids are spared, emaciated adults show dramatic depletions of the middle and deep layers, and upper blubber adipocytes undergo the least metabolic change relative to their importance to streamlining and insulation [47]. Although influenced by environment and species [126], dynamic changes in blubber across seasons and lifespans may affect healing. Across seasons, its lipid composition and fatty acid profiles change [130]. Dolphins, porpoises, and seals show significantly thicker blubber in winter than in summer [133,134], except for some finless porpoise [126]. There are sex differences in blubber thickness [134,135] except for some finless porpoise [126]. Across a lifespan, blubber changes in morphology [133,134] and lipid content [47]. Yearling bottlenose dolphins have significantly thicker blubber than 2- to 12-year-olds [133]. From fetus to adult, blubber mass and depth increase proportionately with body mass and length, and lipid content increases dramatically, such that juvenile and adult blubber have significantly higher lipid content than fetuses; this is due to significant increases in mean adipocyte size rather than cell numbers [47]. In all but massive deep-tissue excisions, blubber thickness correlates inversely with wound severity. For instance, two shark bites penetrating dolphin skin to the same 25 mm depth will create a lesser wound where the blubber is thick on the dorsum and a greater wound where the blubber is thin on the side of the head. Blubber sometimes exudes from wounds as strings or bulges [12,136,137], including an eyewitness account of blubber actively exuding within 7 min of a shark bite [12] and for at least the next 20 min (Figure 8). Blubber could aid healing by providing adipose stem cells [1,46,97]. Despite several studies on its function, development, and morphology, little is known about how blubber itself heals or its potential contributions to wound healing [46].

### 1.7. Natural Antimicrobials

Research on natural dolphin antimicrobials is limited, and potential roles in healing are unknown but beguiling. Marine mammal immunity patterns are like domestic mammals, humans, and rodents but also likely to possess unique immunological features vis-à-vis the spectra of marine microbial pathogens [101,106,118]. The marine environment is a rich source of bioactive compounds [138]; prospecting for new marine antimicrobials is a major driver of drug and biomedical research [139]. Salt water itself has limited antimicrobial properties by killing certain bacteria through osmosis [140], which is why gargling with salt water prevents oral infections.

Dolphin skin contains several antimicrobial compounds that could aid healing, but few empirical data are available. Isovaleric acid could provide antimicrobial protection [1]. Its presence in melon and mandibular fats of delphinids, phocoenids, and monodontids is essential for sound transmission and reception [131]. It is also present in remarkable but uneven quantities in blubber, most concentrated in blubber adjacent to the dermis [131]. Its functional significance in blubber is unknown but may keep it pliant [131]. It is produced endogenously during gestation and increases with age. In humans, it can be toxic [131,141,142] but is used in pharmaceuticals and flavor-enhancers. It is an effective antimicrobial against oral infections [143] and its potential as a natural herbicide, fungicide, and rodenticide is under investigation [144]. Humans use its plant-based counterpart, valerian, as popular homeopathic sedatives, hypnotics, antispasmodics, and hypotensive agents to lower blood pressure [145,146].

Organohalogens in blubber, which bioaccumulate in dugong (*Dugong dugon*) and dolphin blubber from anthropogenic pollution and natural sources [147], could be another natural antibiotic [1,148,149]. Halogenated pharmaceuticals are important in several classes of human antibiotics [147], present in 25% of licensed drugs [150], and usually hailed as “blockbuster drugs” [151].

Dolphin platelet-rich plasma (PRP) and adipose stem cells (ASCs) modulate the immune system through multiple mechanisms [152] and contain innate antibacterial properties that together may provide exciting combination therapies for regenerative medicine in bottlenose dolphins [46]. PRP releases growth factors with antimicrobial properties [153,154] that accelerate wound recovery time [46] and exert anti-inflammatory effects that may reduce pain [155]. Dolphin PRP induces ASCs proliferation and phagocytosis, which is crucial to inflammatory host defense.

Dolphins lack sweat and sebaceous glands, adaptations that required a departure from terrestrial mammal healing models. Human glands secrete natural antibiotics. Human sebum can function as antibacterial and antifungal pheromones [156,157] and sweat glands excrete antimicrobial peptides, including dermcidin, lactoferrin, and cathelicidin [157]. Cathelicidins are broad-spectrum antimicrobials in many vertebrate innate immune systems [158]. They are stored in the secretory granules of neutrophils and macrophages, released extracellularly upon leukocyte activation. The roles of these proline-rich antimicrobial peptides (PrAMPs) in pathophysiology have been studied in mice and humans. The artiodactyl immune system (cattle, buffalo, pig, sheep, goat, deer) includes PrAMPs with antibacterial components, and though data on their expression are limited [158], dolphins are artiodactyl congeners. Mardirossian and colleagues [159] discovered PrAMP Tur1A in bottlenose dolphins, whose potent antibacterial activity could be exploited in the fight against pathogens.

External substances may also play a role in dolphin healing. Intriguing first-time observations of Red Sea Indo-Pacific bottlenose dolphins (*Tursiops aduncus*) repeatedly rubbing skin lesions against gorgonian corals (*Rumphella aggregate*), leather corals (*Sarcophyton* sp.), and sponges (*Ircinia* sp.) are self-medicating with external antibiotics [160]. These species contain 17 bioactive metabolites that are useful for prophylactic treatment of microbial infections.

Potential roles of isovaleric acid, organohalogens, platelet-rich plasma (PRP), adipose stem cells (ASCs), alternatives to human sweat gland exudate, and external antibiotics in dolphin wound healing remain to be explored.

### 1.8. Cutaneous Wound Healing

“Injury” is a broad term for any bodily harm. A “wound” specifically refers to a breach in the natural continuity of skin [141]. Wound depth is defined anthropocentrically. In humans and lab animals, superficial partial-thickness wounds penetrate the epidermis, deep partial-thickness wounds penetrate the epidermis and some dermis, and full-thickness wounds penetrate to muscle [97]. In mammalian wound healing studies, a popular wound model is a 4 mm excision. In humans and lab animals, this creates a full-thickness wound [14]. In dolphins, this creates a superficial partial-thickness wound that barely penetrates the dermis.

Faithful Canvas wounds [12] are characterized by source when known (e.g., shark bites, Figure 3, Figure 6, Figure 7, Figure 8, Figure 9, Figure 10, Figure 11, Figure 12, Figure 13, Figure 14, Figure 15, Figure 16, Figure 17, Figure 18, Figure 19, Figure 20, Figure 21, Figure 22, Figure 23, Figure 24, Figure 25, Figure 26, Figure 27, Figure 28, Figure 29, Figure 30, Figure 37, Figure 38, Figure 39 and Figure 40; fishing line entanglement Figure 4 and Figure 5; propeller blades Figure 41, oystershell slashes, punctures) or as nebulous when the source is unknown (e.g., slashes, gouges Figure 31, Figure 32, Figure 33, Figure 34, Figure 35 and Figure 36). Wounds are further characterized by the degree of wound edge wrenching or apposition. Gaping wounds with wrenched-apart, degloved, or poorly apposed edges are called gnarly wounds in Faithful Canvas terminology; most are also full-thickness (Figure 3A). These wide, deep wounds penetrate epidermis, dermis, blubber, and sometimes muscle, expose underlying tissues, and heal by secondary intention to gnarly scars via granulation followed by reepithelialization. At sea, full-thickness wounds that expose blubber are readily identified (Figure 8 and Figure 27). In contrast, slice wounds with well, closely, or tightly apposed edges are called surgical wounds (Figure 3C) in Faithful Canvas terminology; they heal by primary intention and reepithelialization, forming surgical scars. At sea, the well-apposed wound edges of surgical slices hide underlying tissues, making it difficult to distinguish superficial from deep partial-thickness wounds. Without known macroscopic markers of the amount of blunt force trauma imposed, partial-thickness wounds in the Faithful Canvas collection [12] are labeled tentatively as superficial or deep after observing the healing process. Complete wound healing is defined as full restoration of tissue integrity [161], forming a continuous barrier and restoring normal physiological and anatomic function by regeneration (i.e., original tissue is restored) or repair (i.e., original tissue is replaced by relatively inert scar tissue). In humans, defining clinical success as ‘restoring normal physiological and anatomic function’ needs review [2].

Current knowledge of cutaneous wound healing is constrained for four main reasons. One, healing knowledge is based on limited lab animal models, mostly loose-skinned rodents and rabbits [97], as well as fruit flies (*Drosophila* sp. [162]), zebrafish (*Danio rerio* [163]), frogs and salamanders [14], and tight-skinned human and porcine models. Pigs provide a better model of human healing than rodents [97], but poor genetic tractability, complicated anesthesia and surgical procedures, cost, and housing issues with pigs have promoted the more experimentally tractable rodent models, largely mice, to fundamental models of cellular and molecular mechanisms underlying mammalian tissue repair [2]. Loose-skinned lab animals have very different skin and muscular adaptations from tight-skinned humans and pigs [97]; e.g., rodents have panniculus carnosus muscles that close wounds by contraction in hours vs. days [6] and have a smooth dermal–epidermal junction [117]. Correspondingly, human healing is accurately informed by rodent models roughly 50% of the time and by pig models 75% of the time [97]. The paucity of animal models that closely mirror the human condition complicates transitions from experimental models and preclinical studies to clinical trials [2]. Ergo, new animal models that accurately mimic human acute and chronic wound pathologies are desperately needed [164], especially from adult animals that naturally heal tissues [2] or provide data on wound healing and tissue regeneration in tight-skinned mammals, which Lin et al. [117] labeled of utmost importance. Dolphins present a new healing model independent of the crucial contributions of hair follicles and glands to terrestrial mammal healing. Two, “wounds” are difficult to model and cross-reference due to notable variability in size, depth, history of wound care, co-morbidities, genetic modifications, etc. [97]. These differences make it harder to compare whether dolphins heal faster. Comparisons of healing phase durations and specific chemical mechanisms are further constrained by experimental wound differences, e.g., incisions versus excisions [161]. Three, species and breed differences matter. The interaction between wound severity and species’ morphological, histological, and immunological differences exerts substantial effects on healing [6,22,23,97]. For example, closely related horses and ponies (*Equus ferus caballus*) show considerable differences in second intention wound healing [165]. Pony inflammation is strong but short-lived, leading to better-quality healing. Horse inflammation is weak and chronic, leading to purulent exudate (pus), poorly vascularized exuberant granulation tissue, and more intense scarring. Four, with respect to informing healing at sea, a major departure is that medical models are based on sterile conditions [166] that are wholly antithetical to microbial-rich seas. Open wounds are microbial depots with significant implications for inflammation until closure.

Wound healing happens from the inside out and from the bottom to the top. Healthy healing involves a complex series of tightly orchestrated biological processes, broadly described as the succession of three overlapping phases: inflammation, proliferation (repair or tissue formation), and tissue remodeling (or maturation) [92]. Inflammation fights microbial invasion. Proliferation builds fresh blood vessels at the wound site (angiogenesis), deposits matrix that replaces damaged subepidermal cells (granulation), and recruits keratinocytes to replace damaged skin (reepithelialization). Remodeling eventually creates mature scar tissue. However, borders between these phases are fluid, and overlap is extensive [167,168]. Wound phase partitions are mostly established from lab animals and are mainly useful for instruction [97]. The partition idea undermines the actual continuity and heterogeneity of healing (Figure 39); e.g., one portion of a wound remains in the inflammatory phase after another has progressed to the proliferative phase [97]. Phase overlap probably has a bigger impact on large wounds [97] and on dolphins with multiple wounds, whether from shark bites or propeller slashes (Figure 8, Figure 9, Figure 10, Figure 11, Figure 12, Figure 13, Figure 14, Figure 15, Figure 16, Figure 17, Figure 18, Figure 19, Figure 20, Figure 21, Figure 22, Figure 23, Figure 24, Figure 25, Figure 26, Figure 27, Figure 28, Figure 29, Figure 30, Figure 31, Figure 32, Figure 33, Figure 34, Figure 35, Figure 36, Figure 37, Figure 38, Figure 39, Figure 40 and Figure 41). Further complexity in healing arises from the myriad chemical redundancies that ensure the correct signals and chemotaxis are stimulated at the appropriate times [2,92,97,161].

#### 1.8.1. Wounding

A wound immediately upsets cellular equilibrium. The local microenvironment is flooded with dead, dying, and ruptured cells, which spill their contents (e.g., DNA, mtDNA, RNA, ATP, metabolic products, positive and negative ions), disrupting the oxygen, pH, and ion levels of surrounding cells [168]. Blood vessels leak water, salt, and protein, forming a transudate that causes local swelling (edema). Damaged tissue allows infectious microbes, such as viruses and bacteria, to invade sterile tissues, where they encounter inflammatory antimicrobial agents that clean the wound site and prevent infection. This cellular carnage is death by necrosis, which always triggers inflammation [169]. Innate immune responses are activated as soon as cellular carnage is detected [118,168].

#### 1.8.2. Inflammation

Inflammation is the first healing phase. Its role is to control bleeding, prevent infection, and cleanse the wound, allowing damaged tissues to be replaced. It is sometimes subdivided into vascular (hemostasis) and cellular (inflammatory) responses [97]. Hemostasis stops the bleeding, and the blood vessels vasoconstrict. Blood is transformed from a liquid to a gel by pro-coagulants and prothrombin, forming a fibrin clot of collagen, thrombin, fibronectin, and platelets. Endothelium and nearby platelets aggregate at the wound, start the coagulation cascade (markedly slowed in cetaceans [52]), and release factors that attract the necessary cells for subsequent healing.

Griffeth and colleagues [46] were the first to quantify dolphin platelet growth factors. Dolphin platelets are larger than human platelets and release several substances that promote tissue repair, angiogenesis, and inflammation. Platelet-derived growth factor (PDGF) shortens the recovery time of open wounds. Clinically harvested as platelet-rich plasma (PRP) by concentrating platelets, PRP has been used clinically for years in regenerative medicine in multiple mammals, including humans [46]. PRP occurs in comparable quantities in humans and dolphins. The PRP growth factor vascular endothelial growth factor (VEGF) is fundamental because healing requires the reestablishment of a functional vascular network (angiogenesis), and VEGF is among the most potent pro-angiogenic agents. VEGF levels are significantly lower in dolphins than in humans [46]. The PRP growth factor transforming growth factor beta (TGFβ) is a potent component that stimulates fibroblast production and migration to the wound (which stimulates collagen and ECM-rich matrix proliferation); triggers fibroblast differentiation into myofibroblasts, which promote wound closure; and releases tissue inhibitor of metalloproteinases (TIMP) secretions that decrease production of destructive matrix metalloproteinases (MMPs). Although a potent healing promoter, excess TGFβ may lead to overhealing outcomes, including hypertrophic and keloidal scars [170]. Alternatively, knockdown of the TGF-β1 signaling axis reduces scarring [2]. TGF levels are significantly lower in dolphins than in humans [46]. Finally, where platelets occur, adipose stem cells (ASCs) are also present [46]. Clinically, PRP and ASCs are used separately or together to successfully regenerate human soft tissue defects, scars, and burns [171]. Bottlenose dolphin adipose stem cells were first isolated in 2012 [172]. They meet all criteria for mesenchymal stem cells, including differentiating into adipocytes (cells in connective tissue specialized for fat storage), osteocytes (bone-forming cells), and chondrocytes (cells that produce and maintain the extracellular matrix of cartilage, which is composed of collagen and the proteoglycans that bind to water molecules and increase cartilage’s resistance to compression). In vitro PRP-treated stem cells proliferate at significantly increased rates. In vivo undifferentiated stem cells delivered by platelet-rich plasma migrate to the wound site and trigger proliferation [46]. In nature, dolphin adipose stem cells may play a role in regeneration [46].

Inflammation intensifies cellular traffic to the wound. This is expedited by vasodilation of nearby blood vessels, which increases vascular permeability, and the fibrin clot, which serves as a loose scaffold through which incoming neutrophils, macrophages, and fibroblasts migrate to the wound [92]. Dolphins form a clot to stop bleeding but do not form a hard scab [15].

Platelets in fibrin clots also generate cellular signals that induce the first inflammatory cells to migrate to the wound, neutrophils, which sanitize the wound by consuming cells (phagocytosis) and debriding debris [118]. Neutrophils are part of the innate inflammatory response (also known as acute-phase [169]). Innate inflammation is well equipped to engulf microbes but is relatively indiscriminate in its responses [118]. It involves white blood cells (neutrophils, eosinophils, and macrophages), natural killer lymphocytes (NK cells) [173], and antimicrobial factors (e.g., complement, lysozyme, lactoferrin, defensins, and reactive oxygen and nitrogen intermediates [118]). Although epithelial cells and fibroblasts can phagocytose antigens to some extent, and keratinocytes and melanin are avid phagocytes [113], the “professional” or dedicated phagocytes are immature dendritic cells (DCs) and the inflammatory monocytes, macrophages, and neutrophils [174].

Neutrophils are major, primordial pathogen-fighting immune cells that disinfect lifeforms from slime molds to mammals [175]. They are the professional phagocyte ‘first responders’ [174]. Their central functions are the ability to be recruited to wounded or infected sites and to perform antimicrobial services [175]. In vertebrates, they are produced in the bone marrow and released into the bloodstream, where they travel to wherever they are needed. The mature form has a structural specialization, a poly-lobed nucleus, that facilitates mechanical deformation and allows neutrophils to “squeeze” through tight spaces when migrating to the wound [174]. Neutrophils are equipped with a vast array of antimicrobial mechanisms stored in abundant amounts in granules [118,174] to help them recognize, engulf, and kill large (>0.5 μm) pathogens by phagocytosis [175]. Their extensive array of microbe-killing mechanisms includes iron-withholding molecules [174], the discharge of nuclear contents to form neutrophil extracellular traps, and the release of antimicrobial peptides [175]. They clear wound sites by releasing several caustic enzymes (proteases) that digest bacteria and necrotic tissues, and by generating reactive oxygen free radicals that help sterilize the wound. Neutrophil proteases and matrix metalloproteinase (MMP) clear damaged extracellular matrix (ECM) from the wound area. In dolphins, neutrophils dominate the first days of inflammation, seconded by eosinophils [15].

Should pathogens overwhelm innate inflammation, a second inflammatory response called the adaptive [118], acquired [169], or antigen-specific [176] immune response is triggered. It involves activated B- and T-lymphocytes, B-lymphocyte antibodies (immunoglobulins), and dedicated natural killer (NK) cells [173] that fight infection by targeting and neutralizing pathogens such as viruses and bacteria. Unlike rather indiscriminate innate inflammation, the antimicrobial agents of adaptive immunity are highly antigen-specific [118] and improve their selective responses over time via immunological memory [169]. However, little is currently known about adaptive immunity in mammal healing [2]. Recent discoveries of γδ T cells, located predominantly in the dermis and blubber among stranded Fraser’s dolphin, revealed an increased presence in healing wounds that suggests connections with healing rather than pathogen defense and that γδ T cells may act as a crucial link between innate and adaptive immune systems [177].

If microbial contamination has not recurred, neutrophil invasion in lab animals will cease within a few days [14]. Neutrophils live fast and die young. They phagocytose antigens for 6–12 h before receiving chemical signals to end their destructive activities and undergo programmed cell death (apoptosis). Unlike necrosis, neutrophil apoptosis does not trigger further inflammation because their devitalized cell contents are released in packets called apoptotic bodies that confine their pathogenic contents [169]. Then, in a complementary strategy that further minimizes the risk of accidental recontamination, neutrophil apoptosis is synchronized with monocytes in nearby tissues and blood that migrate to the wound, transform into macrophages, usually 48 to 96 h after injury (2–4 days), and begin replicating [92]. The antimicrobial capacity of neutrophils is higher than that of macrophages, and the phagocytosed cargo they ingest is highly pathogenic [174]. The much larger macrophages engulf the pathogenic contents of smaller devitalized neutrophils and dispose of them safely without renewed cytotoxic exposure to surrounding tissues [174].

Macrophages are another professional phagocyte. Broughton [92] called them “maestros” because they orchestrate inflammation and proliferation, first inducing and then restraining inflammation by exerting both pro- and anti-inflammatory influences. Macrophages trap, engulf, and destroy microbes. These chemical maestros also maintain and amplify inflammation by secreting proinflammatory cytokines (interleukin-6 (IL-6), IL-1, and tumor necrosis factor alpha (TNFα)). Activated macrophages orchestrate the transition into proliferation by providing growth factors that stimulate fibroblasts, myofibroblasts, and endothelial cell proliferation (PDGF, TGFα, TGF, insulin-like growth factor IGF-1 (and fibroblast growth factor FGF [14]) and promote the construction of new blood vessels (angiogenesis, VEGF), extracellular matrix (ECM), and fibroplasia (scar formation) [2,168]. ECM also plays essential roles in the efficient delivery of growth factors for the induction of blood vessel growth [2]. The advent of macrophages marks the onset of tissue formation and proliferation [178].

One of the key events signaling the waning of inflammation is a phenotypic switch in recruited macrophages from pro- to anti-inflammatory. Anti-inflammatory macrophages release anti-inflammatory cytokines and growth factors that send lipoxin “stop” signals, promoting macrophage polarization toward an anti-inflammatory state and inhibiting neutrophil infiltration [92]. This switch requires delicacy and discretion. Unless the destructive processes of inflammation are balanced and then offset by the constructive processes of proliferation [92], ongoing exuberant inflammation hinders healing [166]. Exuberant inflammation creates chronic (unhealing) wounds, which in humans have reached epidemic proportions in Europe and America [2].

#### 1.8.3. Proliferation

The role of proliferation is to restore damaged tissues. The proliferation of lesions from dermatitis outbreaks on stranded carcasses [90] and live free-ranging dolphins [179,180,181] is a separate process addressed elsewhere [Weaver, in prep.]. In both dolphins [15] and humans [92], proliferation typically begins 3 days after injury. It repairs damaged blood vessels (angiogenesis), fills the wound lumen with granulation tissue, and replaces damaged skin by keratinocyte-mediated reepithelialization [182]. The body repairs damaged tissues by replacing or repairing them. In replacement, damaged epidermis is restored like new without a scar; this is reepithelialization. It is the main type of healing in well-apposed wounds and follows granulation in poorly apposed wounds [97]. Reepithelialization involves keratinocytes produced in the proliferative dermal–epidermal junction (Figure 1) that move to the skin surface. In a controlled wounding study of dolphins [15], naturally high baseline mitotic rates along the proliferative dermal–epidermal junction accelerated a dramatic nine-fold increase between 2 and 7 days of healing, fully restoring the epidermis by day 3 (reepithelialization) and dermis by day 7; note that experimental wounds were superficial. Keratinocytes move to the skin surface independently [19]. This cellular asynchronicity is magnified in bottlenose dolphins by the extreme folds of their dermal–epidermal junction, in that cells at dermal papillae tips ascend a short distance in a straight line to the surface (stratum externum). In contrast, cells along the sides of the dermal papillae must move laterally and then migrate upward, taking longer to reach the externum [103]. Bottlenose dolphin epidermal cells reach the surface an average of 73 days after leaving the basale layer. This is 1.7 times longer than the 40- to 45-day transit time for human epidermal cells; however, in dolphins, it is 8.5 times faster due to marked differences in skin thickness. Dolphin cells migrate an average distance of 1.2 mm in 73 days, whereas human cells migrate 0.1 mm in 45 days.

In repair, damaged tissue is not restored; scar tissue takes its place. Following the fibrin clot, repair involves a provisional matrix, granulation, and scar tissue. Early in wound healing, the provisional matrix is composed mainly of fibrin and fibronectin (a structural ECM glycoprotein that facilitates cell adhesion). This haphazard, disorganized collection of glycans is thin and compliant, allowing the now-intense cellular traffic of inflammatory neutrophils, lymphocytes, macrophages, and proliferative fibroblasts to move through it easily. In the provisional matrix, wound fibroblasts are stimulated from their quiescent state to migrate into the wound by growth factors and cytokines, mainly from platelets and activated macrophages, where they begin synthesizing collagen III. The provisional matrix promotes granulation (filling the wound), stimulates wound contraction to seal wound edges [92], and provides the preliminary tissue scaffolding that is eventually replaced by mature scar tissue. Granulation tissue is new connective tissue thick with sprouting blood vessels, macrophages, fibroblasts, ECM, fibronectin, elastin, proteoglycans, hyaluronic acid, and collagen III [92,97]. It repairs the open space or lumen of deep wounds by filling it from the bottom and sides [97].

In humans and lab animals, granulation tissue begins to form in the wound bed approximately 4 days after injury [14], replacing the papillary dermis the fastest, followed by the mid-dermis and the lower reticular dermis [97]. In terrestrial mammals, granulation islands develop from hair follicle bulbs and grow individually until they merge with other islands [183]. In mouse models, hair growth patterns across different skin regions significantly affect skin healing rates [2]. The loss of cetacean hair is an adaptation that requires a departure from the terrestrial mammalian healing model of granulation islands generated by hair follicles.

Granulation tissue is stratified in four layers [184]. The bottom 50% is the fibroblast layer, overlaid by a capillary layer with many inflammatory cells (25%), then loose connective tissue (15%), and finally slough (10%). As such, granulation is mainly composed of fibroblasts. Fibroblasts produce collagen, ECM, and additional granulation tissue, while stimulating keratinocytes to reepithelialize [92,97]. The most important growth factor for fibroblast proliferation is platelet-derived growth factor (PDGF) [46,171]. Fibroblasts already located in the wound site, called wound fibroblasts, also begin synthesizing collagen. Exposure to TGFβ and mechanical loading signals transform a portion of fibroblasts into contractile specialists called myofibroblasts, which contract, pulling the wound closed [2]. In granulation tissue, the type and number of inflammatory cells depend on its stage of development and the presence of antigens but are mainly macrophages [97].

Early collagen is mostly collagen III, whose fibers are oriented parallel to the skin instead of the uninjured “crisscross” basketweave pattern and whose appearance coincides with the cellular adhesive fibronectin. During proliferation, the thinner and more flexible collagen III fibers predominate in granulation tissue. Once granulation has filled the wound, fibroblasts synthesize and secrete keratinocyte growth factor (KGF), which stimulates neighboring keratinocytes to migrate to the wound site, proliferate, and reepithelialize (cover with new epidermis) the developing scar tissue. Keratinocytes also stimulate angiogenesis by secreting VEGF. In healthy healing, these chemical processes are gradually turned off.

The main study on live bottlenose dolphin immunity at sea was Bruce-Allen and Geraci’s controlled wounding study on three adolescent female dolphins housed in sea pens [15]. The authors created superficial partial-thickness wounds (six 10 cm-long, 2 mm-deep surgical incisions) in the epidermis. For histology, they biopsied wound surfaces at 2, 6, and 12 h and 1, 2, 3, 7, and 10 days post-injury. Swelling was immediate and visible for days. Within two hours, coagulated blood was visible at the base of the wounds, but no blood could be elicited after two hours; the dolphin wounds did not develop the hard scabs of terrestrial mammals. By six hours, inflammation was underway with an influx of 90% neutrophils. From 6 h through to 3 days, the wounds were covered with a loose layer of degenerating mixed cell infiltrate predominated by inflammatory neutrophils, intraepidermal vesicles, and necrotic cells (i.e., the previously noted cellular carnage), which in the absence of hard scabs the authors characterized as a protective buffer zone that functioned like a hard scab. By day 1, pale gray tissue obscured the base of the cuts, and the now-spongy incisional shoulders had a sharp black outline. In Faithful Canvas terms, this is a wound in the bi-colored dark-to-light pigment pattern. Inflammatory cells were restricted to small pockets in the dermis or wound bed. Though restricted, elevated mitotic activity of basale cells on days 2–7 restored full epidermal thickness and layering by day 3 (reepithelialization) and full regeneration of the dermal–epidermal junction by day 7. Melanosomes were present in newly formed epidermal cells but showed an abnormal intracellular distribution; no mention of repigmentation was made. Healing was complete in a week.

#### 1.8.4. Remodeling

The last phase of wound healing is the reorganization of young, immature scar tissue, dominated by collagen III, into stronger, more organized mature scar tissue, dominated by collagen I. During remodeling, initially thin, flexible collagen III fibers are slowly reabsorbed and replaced by thicker, stiffer collagen I fibers organized along stress lines, which toughen the scar tissue [92], making it denser, stiffer, and less compliant. Fibroblasts adapt to the changing mechanical loading on the scar as it matures; isometric tension develops as internal and external mechanical forces are balanced. Remodeling tentatively normalizes epidermal thickness and cellular content, extracellular matrix composition, and blood vessel count as close as possible to the pre-wound stage [97]. Depending on wound severity, the result can range from very close to the unwounded skin (Figure 19, Figure 31 and Figure 36) to severely altered, functionally and cosmetically (Figure 13, Figure 26, Figure 30, Figure 37 and Figure 40).

On the continuum of skin trauma outcomes, one end is regeneration (rare in the animal kingdom) and the other end is scarring (the usual outcome of mammal wounds). Scarring is a sign of regenerative failure or pathological connective tissue [2]. Scars are made of collagen [92]. Scarring reflects the body’s inability to reassemble collagen into its original basketweave pattern, strength, and flexibility, although in most cases, scars restore normal function [113,185]. Macroscopically, there are four types of scars. Fortuna scars are flush with the uninjured skin surface. Hypertrophic scars rise above the skin surface but eventually flatten. Keloidal scars extend far above and beyond the original wound. In contrast, atrophic scars are sunken below the skin surface because scar tissue fills the wound lumen incompletely [186]. Unlike terrestrial mammals, most dolphin scars are atrophic (Greek for “wasted” [12]). Human atrophic scars have three subtypes: ice pick scars (60–70% of atrophic scars), rolling scars (15–25%), and boxcar scars (20–30% [187]. Whereas human scar tissue becomes more refined and less visible over time [97], most dolphin scars remain atrophic (Figure 7) and most resemble human boxcar scars [12].

A scar’s integrity, stability, and restoration of function depend on interactions between its tissue volume and organization, resident cell populations, and quality of deposited connective tissue [185]. In humans and lab animals, collagen levels are different in unwounded, healing, and remodeled skin [92]. Unwounded skin is 80–90% collagen I and 10–20% is collagen III. Healing granulation tissue is 30% collagen III. In remodeled mature scars, collagen III is back down to 10%. Preliminary understanding of percentages of collagen III to I in dolphins suggests that the dolphin ratio follows terrestrial mammals, with lower percentages of collagen III in unwounded skin, relatively higher percentages in granulating healing tissues, and lower levels again in mature scar tissue [23]. The new scar never achieves 100% of the flexibility or strength of uninjured skin [188]. At one week, the wound has only 3% of its final strength. At 3 weeks, it is 30% of its final strength. By 12 weeks and beyond, it is approximately 80% of its final strength. In dolphins, curtailed strength and flexibility raise questions about the resumption of aerial behaviors, especially 3–12 weeks post-wounding (Figure 6), because the flexibility required for leaping out of the water is limited by scar tissue [36]. Even after maturing for a year, scar collagen will never become as organized as collagen in uninjured skin [92]. Moreover, collagen disarray and variations in fiber thickness may be normal for bottlenose dolphins, and, as in harbor porpoises (*Phocoena phocoena*), may show greater density and diameter in less flexible areas of the body, with fiber orientation conforming to local needs for flexibility [189]. Although many human and lab animal scars remodel for months [14], dynamic changes (Figure 39 and Figure 40) show that many dolphin scars remodel for years [12]. To the best of our knowledge, this is the first study to document macroscopic healing-related pigment patterns on free-ranging bottlenose dolphins, visible to boat-based observers, in 106 detailed, longitudinal healing histories [12], and to link them to the underlying histology of inflammation, proliferation, and remodeling.

## 2. Materials and Methods

**Figure 2:**
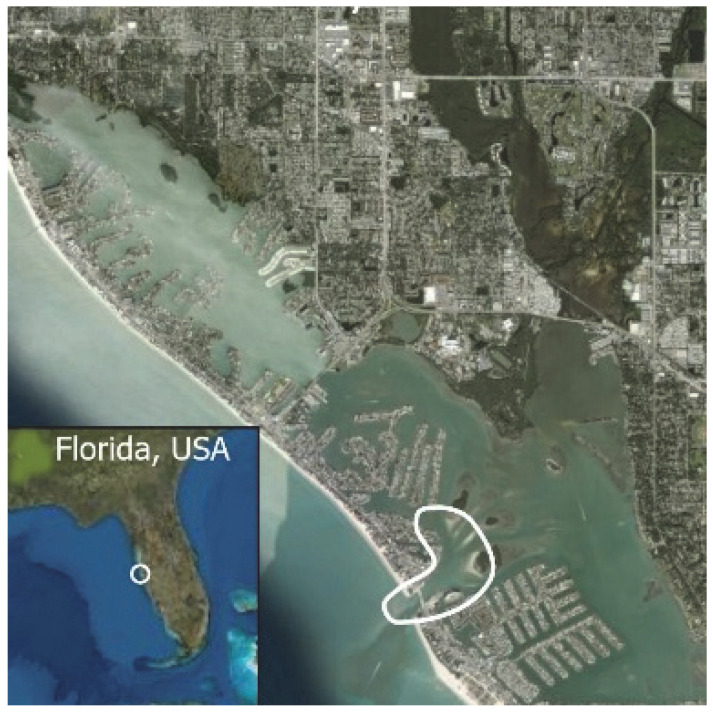
John’s Pass Intracoastal Waterway study area (north end: 27.831986° −82.830557°, south end: 27.771542° −82.753089°) from Redington Shores to Treasure Island, Pinellas County, Florida, USA. White outlines the John’s Pass construction zone (John’s Pass (27.782679°, −82.782705°)). John’s Pass dolphins are part of the Tampa Bay, Florida, stock.

AW conducted an intensive ethological study from 2004 to 2024 on population, behavioral, and wounding patterns of free-ranging bottlenose dolphins (*Tursiops truncatus*) encountered in the John’s Pass study area, west central Florida, St. Petersburg, FL, USA (Figure 2), under NOAA permits 1088-1815, 16299, 20346, and 25957. The sampling effort consisted of 1–4 surveys of the study area/week/20 years, *N* = 1831 surveys, logged approx. 24,000 survey miles, *N* = 474 identified dolphins. AW recognized 400+ dolphins at sea on sight, collected all behavioral data and photos, and wrote the healing histories and scar searches based on extensive direct observations and scrutiny of her 950,000-photo archive [12]. USCS Captain John Heidemann drove the boat and helped spot. All dolphins were identified by dorsal fin notches and body scars documented with mark-recapture photo identification [190] with high-speed 20D, 50D, and 80D Canon digital cameras and a 70–200 mm digital zoom lens. The best photos of each dolphin were dated, labeled, stored by survey, and logged into the Gulf of Mexico Dolphin Identification System (GoMDIS OBIS-SEAMAP).

Data were collected during small-boat surveys of the John’s Pass study area (Figure 2), a narrow 10 km section of the contiguous Boca Ciego Intracoastal Waterway (north end: 27.831986° −82.830557°, south end: 27.771542° −82.753089) of the west side of St Petersburg, FL, USA peninsula. The white bean shape (Figure 2) outlines the John’s Pass construction zone, ground zero for this study [12,25,36,53,191,192,193,194,195]. The sea floor is a patchwork of hard-packed sand, muddy sand, oyster bars, and continuous and patchy seagrass beds. Water depth averages 1–3 m deep except in John’s Pass, which is 6–12 m deep. The study area was surveyed year-round by a small boat (5.8 m Proline, Yamaha 115 hp outboard) on a line transect between channel markers with good visual access to both shorelines. Surveys started within two hours after dawn or as dictated by the weather. A complete survey started from the home dock (72.785173°, −82.771513°), first stop was John’s Pass (27.782679°, −82.782705°), continued to the north end of the study area, retraced the route halfway but continued east of John’s Pass tidal deltas to the southern end of the study area, and ended back at the home dock. Survey time depended on dolphin sightings. Surveys without sightings (3% of all surveys) lasted an average of 2 h. Surveys with sightings (97% of all surveys) lasted up to 8 hrs. Surveys typically ended between 11 a.m. and 2 p.m. Sampling was homogeneous, as the entire study area was surveyed (except for surveys aborted by bad weather). This study used a sequential research design by collecting observations across the entire study area to compare at multiple points in time [196].

Vessel distance to dolphins ranged from next to at the boat to several hundred yards away. Data for healing histories and scar searches were collected from high quality photos taken with a long lens generally when dolphins were 0–3 boat lengths (0–18 m) away. The criterion for selecting usable photographs was choosing those that provided the clearest illustration of changes across healing. Each dolphin’s healing history was written based on a complete perusal of that dolphin’s entire sampling history and every photograph I had taken of the dolphin. Pigment patterns and other patterns were named and described as they emerged during the derivation of each healing history. As histories accumulated, data verifying pigment patterns and other patterns accumulated. When wound age was unknown, I did not estimate wound age; instead I provided days since I had first seen the wound. Potential sources of measurement error included poor quality photos, low light, photos occluded by glare or not precisely perpendicular to the body location of the wound, and photographic laterality (defined in Faithful Canvas collection terms as a dolphin’s tendencies to present the same (only one) side of their body to the research boat per sighting despite active efforts to photograph both sides of every dolphin encountered); these were excluded from healing histories.

Healing histories characterize the macroscopic features of wound healing, scars, and skin conditions visible to boat-based observers on portions of the dolphin’s body typically above the waterline when it surfaces to breathe. Underwater observations were not conducted; turbidity was not an issue. Wounds and scars were mainly from shark–dolphin interactions, indicated by crescent-shaped wounds [66,74,82]. Compagno et al.’s [197] comprehensive shark review cited great white (*Carcharodon carcharias*), tiger (*Galeocerdo cuvieri*), and bull sharks (*Carcharhinus leucas*) as the most likely dolphin predators; tiger and bull sharks are common Florida sharks. No tissue sampling was undertaken, and no medication was administered. Two of the many limitations to tracking dolphin healing at sea are the influence of photo light quality on the appearance of healing wounds (i.e., overcast days reduce and bright sunny days enhance contrast) and photographic laterality (in which only one side of a dolphin is typically available for photographic documentation per sighting [12]). Sighting gaps also introduce interruptions in healing data. The method of matching Faithful Canvas macroscopic markers of healing to published stages of dolphin healing at sea involved thorough intense scrutiny of published descriptions and photos to identify aspects that did and did not match Faithful Canvas records [12].

## 3. Results

### 3.1. Healing-Related Pigment Patterns

Macroscopic markers from the Faithful Canvas collection [12] are illustrated in Figure 3, Figure 4, Figure 5, Figure 6, Figure 7, Figure 8, Figure 9, Figure 10, Figure 11, Figure 12, Figure 13, Figure 14, Figure 15, Figure 16, Figure 17, Figure 18, Figure 19, Figure 20, Figure 21, Figure 22, Figure 23, Figure 24, Figure 25, Figure 26, Figure 27, Figure 28, Figure 29, Figure 30, Figure 31, Figure 32, Figure 33, Figure 34, Figure 35, Figure 36, Figure 37, Figure 38, Figure 39, Figure 40 and Figure 41. They show that dolphin wounds heal in three broad, consecutive macroscopic markers: initial swelling of fresh wounds, the dark-to-light pigment pattern, and the light-to-dark pigment pattern. Pigment patterns are measured from the outside in, towards the center or wound bed. All healing histories show both pigment patterns in the reliable sequence: dark-to-light first, then light-to-dark. Durations of pigment patterns vary across wound severity, body location, dolphin age and general condition, season, and blunt force trauma. Partial-thickness and full-thickness wounds on the body generally seal in 4–8 weeks. Dermatitises and slices into the leading edge of dorsal fins generally take months to heal [12].

#### 3.1.1. Fresh Wounds

**Figure 3:**
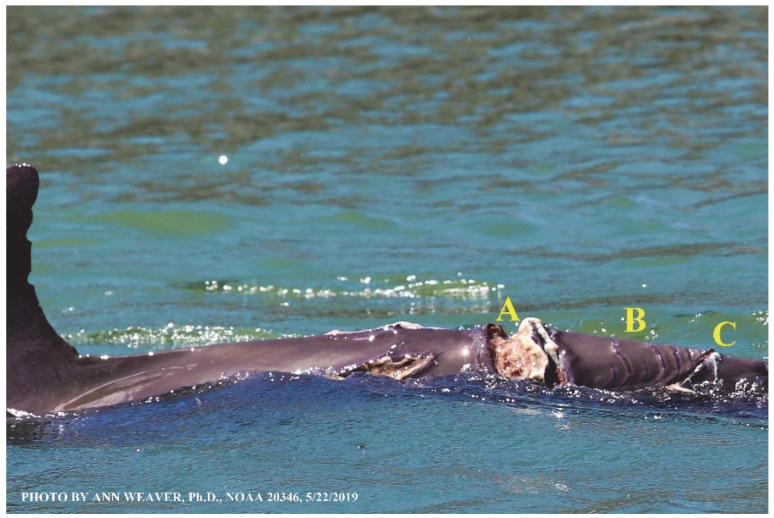
Examples of fresh gnarly (A) and surgical (B,C) wounds, in this case from shark bites. Source: [12] Vol. 1 Introduction, Ch. 2 Glossary, Photo 1, HICS.

Fresh wounds emanate from a range of sources (e.g., sharks, oysters, and stingray spines) [12]. The only Faithful Canvas wound that resembled “core sample” wounds [6,20] was a puncture. Fresh wounds range between gnarly (degloved) and surgical (non-degloved) (Figure 3, Figure 8, Figure 15, Figure 20, Figure 27 and Figure 37). Fresh gnarly wounds are easy to see because they expose underlying pale yellow-beige-pink tissues (Figure 3A). In contrast, fresh surgical wounds are hard to see. For example, shark tooth rakes dragged lightly across dolphin skin are initially inconspicuous, resembling black parallel slices from surgical razors against the dolphin’s dark gray body (Figure 15). Detecting a fresh wound at sea is further impaired by the wounded dolphin’s initial body slamming and agitated swimming. Wound edges are initially sharp but soon become rounded because the body’s first macroscopic response is swollen (edema) edges that outline fresh wounds like faint halos (Figure 8, Figure 20 and Figure 27). Some wounds exude blubber bulges or strings (Figure 8). Swelling and blubber bulging began within three minutes on an eye-witnessed head puncture ([12] Vol. 3 Head Bites, Ch. 12 Keynote Case, Bet) and within 7 min on an eye-witnessed shark attack ([12] Vol. 2 Body Bites, Ch. 2 Keynote Case, Saga).

#### 3.1.2. Dark-to-Light Pigment Pattern

**Figure 4:**
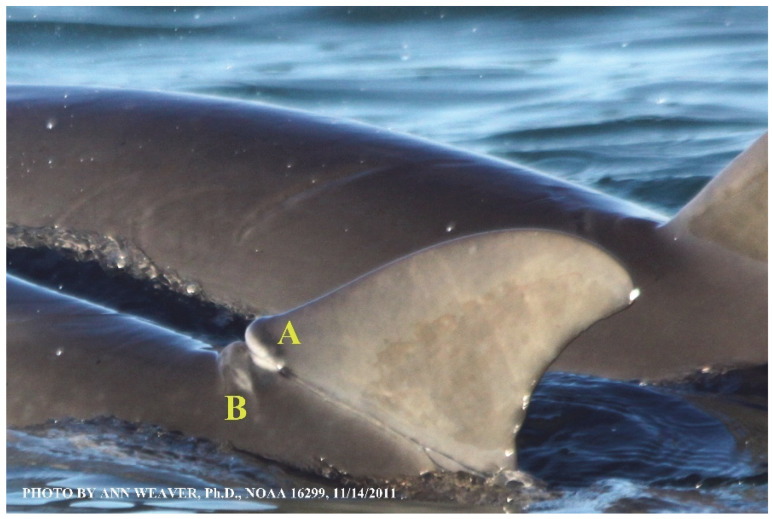
A: Example of the bi-colored dark-to-light pigment pattern on a chronic wound. B: Faded light-to-dark pigment pattern on older wound Source: [12] Vol. 5 Dorsal Fin Slices and Scrapes, Ch. 2 Keynote Case, Vidalia, Photo 29. Vidalia’s estimated age: 284 days/9.5 months; 14 November 2011 (67 °F/19 °C).

The body’s second major macroscopic marker and first pigment pattern to develop is the dark-to-light pigment pattern (Figure 4, Figure 9, Figure 17, Figure 21, Figure 28, Figure 31 and Figure 38). Pigment patterns are color bands called borders labeled from the outside in. “Dark” refers to the hyperpigmented outer border. “Light” refers to the hypopigmented inner border. Fresh, swollen wound edges lack borders and often remain swollen well after pigment pattern development. The dark-to-light pigment pattern generally develops visibly 1–3 days after wounding and usually lasts 4–8 weeks. The inner hypopigmented or white border develops first and outlines the wound. One or two days later, the outer hyperpigmented or dark border develops, outlining and accentuating the inner white border. The dark-to-light pigment pattern can be bi- or tri-colored depending on the degree of degloving. “Bi” refers to two borders, outer and inner (Figure 9A), which develop on surgical wounds whose edges are too close together or well-apposed (not degloved) to expose underlying tissue. “Tri” refers to three borders: outer, inner, and third inner ‘border’ of exposed underlying tissue (Figure 9B), which develop on gnarly wounds whose edges were wrenched apart or poorly apposed (degloved) enough to expose underlying tissue. Some inner white borders show a ‘brightening’ by changing color from light gray (Figure 16), to bright white (Figure 17), to dull white, and eventually assuming the light gray halo of the second pigment pattern (Figure 18).

The outer dark border is well developed by about a week, although its color intensity and breadth vary more than those of the white inner borders. Dark borders may be complete or fragmented (white borders rarely fragment). Dark outer borders are thinnest or faintest on well-apposed wounds and fin wounds. They usually develop more conspicuously on wounds on the body and on poorly apposed, gnarly wounds. The white borders of well-apposed wounds obscure granulating interiors. Poorly apposed wounds expose them. The beige-pink interiors of fresh, degloved wounds give way to pink granulation tissue; the color and vibrancy of tri-colored wounds show dynamic changes from deep to pale pink as granulation intensifies and fades.

Granulation fills the wound lumen from the bottom and sides, completing this task first at the best-apposed wound edges as paths of least resistance (Figure 28). On crescent-shaped shark bites, the location of the least-apposed wound edges shows where the shark bite wrenched skin the most. The amount of granulation needed to heal a single asymmetrical crescent wound varies across the wound, with the most in deeper wounds where the edges were wrenched apart the most and the least in well-apposed wounds.

#### 3.1.3. Light-to-Dark Pigment Pattern Follows the Dark-to-Light Pigment Pattern

**Figure 5:**
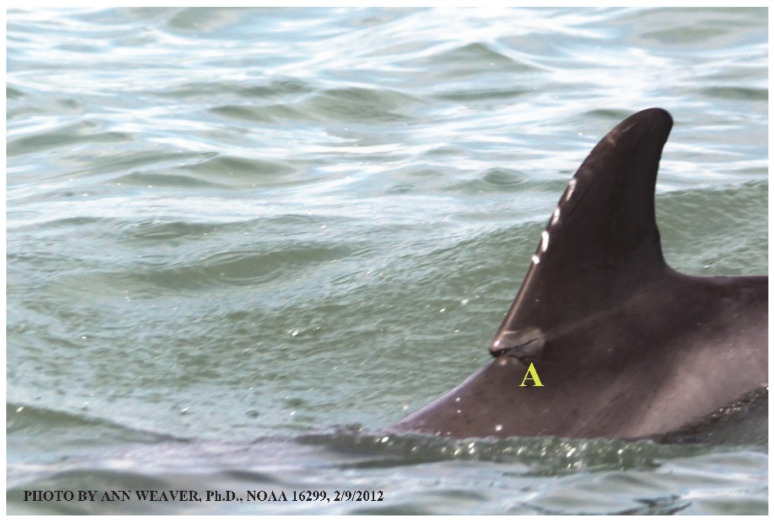
A: Example of the bi-colored light-to-dark pigment pattern. Source: [12] Vol. 5 Dorsal Fin Slices and Scrapes, Ch. 2 Keynote Case, Vidalia, Photo 39. Vidalia’s estimated age: 371 days/1 year; 9 February 2012 (61 °F/16 °C).

After 4–8 weeks, most dolphin wounds have a preliminary seal and have reversed their previous dark-to-light pigment pattern to the opposite light-to-dark pigment pattern. “Light” refers to the hypopigmented outer border. “Dark” refers to the hyperpigmented inner border. New scars in the light-to-dark pigment pattern are also bi- or tri-colored as a function of degloving (Figure 5, Figure 11, Figure 18, Figure 23, Figure 29, Figure 34 and Figure 39). Dolphin cutaneous scars have three basic macroscopic components whose visibility is positively correlated with degloving (ergo, most visible on gnarly scars (Figure 13B)): (1) The outer upper zone is wound/scar edges; they can be smooth or irregular. On scars, wound edges resemble the faint halo of a fresh, swollen wound and fade over time. (2) The middle zone is the tissue escarpments that connect wound edges to the wound bed. Hyperpigmented tissue escarpments create the inner dark border and eventually develop perpendicular, parallel rung-like striations, similar to Su et al.’s [6] constriction lines. (3) The interior zone is the riverine scar marking the wound bed. Many scars stretch by widening over time, so riverine scars undergo riverine shifts. On tri-colored scars, the interior is a winding hypopigmented riverine scar that slowly repigments to hyperpigmentation. The dynamic features of pigment patterns can be used to broadly age a wound or scar.

### 3.2. Matches Between Different Sources of Macroscopic Markers of Healing at Sea

Three studies described macroscopic markers of healing stages in free-ranging dolphins [6,20,78]. Careful comparison to one another and to Faithful Canvas [12] pigment patterns reveals strong matches. Smith et al. [78] described three stages while tracking shark predation pressure on 262 dolphins of three inshore species of tropical Australian dolphins: fresh (stage 1: open wound, broken skin, visible muscle or blood), healing (stage 2: partially healed, clearly visible, white scarring), and healed (stage 3: healed, faint evidence of scarring). The other two studies described healing stages of “core sample” wounds 25 years apart (Table 2). Weller et al. [20] described five healing stages visible in photographs of surgical biopsies on 16 temporarily restrained free-ranging bottlenose dolphins obtained immediately after surgical biopsy through 476 days/1.3 years post-biopsy. Their surgical biopsy wounds were full-thickness wounds penetrating blubber 30–50 mm wide and 10 mm deep. They defined five stages: fresh wounds, three stages of healing, and healed scars (Table 2). Healing days were known, but sighting gaps hampered detail; e.g., Weller stages 2 and 3 in Table 2 show a gap between 26 and 40 days of healing, a particularly dynamic phase of healing [12]. Su et al. [6,22,23] described five macroscopic markers of full-thickness cookie-cutter shark (*Isistius brasiliensis*) “core samples” out of four freshly stranded (dolphin corpses washed into the shallows) Fraser’s dolphins wounds measured 50–80 mm wide and 20–30 mm deep. Su et al.’s five stages were fresh wounds, two stages of healing wounds, and two stages of healed scars (Table 2). Table 2 shows the close matches in healing stages across studies [6,12,20].

**Table 2:** Descriptions of stages of dolphin healing at sea.

Weller et al. 1997 [20]	Su et al. 2022, 2023 [6,22,23]	Weaver, Faithful Canvas Collection [12]
Stage 0: Fresh wound. Oval-shaped biopsy wounds were immediately deeply pink to red in color with no other apparent coloration.	Stage 1: New wounds. Sharp edges, little bleeding, and intercellular epidermal edema, *n* = 2 wounds.	Fresh wound. Sharp edges, no bleeding, swelling. Figure 3A–C, Figure 8A–C, Figure 15A, Figure 20A, Figure 22A, Figure 27A,B and Figure 37.
Stage 1: Early wound healing. At 8–18 days post-biopsy, wounds were pinkish to white, dark at the center and edges, and surrounded by a lighter gray halo fading into normal skin.	Stage 2: Initially healing wounds without granulation, *n* = 6 wounds.	Bi-colored dark-to-light pigment pattern = Weller’s dark/white borders. Su et al.’s necrotic tissue = inner white border. Figure 4A, Figure 9A,D, Figure 10A, Figure 16A,B, Figure 17, Figure 21B and Figure 38.
Stage 2: Intermediate wound healing. At 15–26 days post-biopsy, wounds were no longer pink. Has an outer hyperpigmented border, a white color with a darker spot at the center, and a lighter gray halo.	Stage 3: Healing wounds with granulation. Neo-epidermis, granulation. melanocytes, *n* = 1 wound.	Tri-colored dark-to-light pigment pattern; pink is granulation. Weller’s surrounding halo/dark center = dark-to-light pigment pattern. Su et al.’s necrotic cell = inner white border. Figure 4B, Figure 9B,C, Figure 10B,C, Figure 21A,C,D, Figure 22D, Figure 28A,B, Figure 31, Figure 32 and Figure 39.
Stage 3: Late wound healing. At 40–42 days post-biopsy, the new scar was a white spot without discoloration or apparent epidermal depression.	Stage 4: Healed wound with cellular and vascular blubber. Restoration of normal skin architecture, *n* = 14 scars.	Bi- or tri-colored light-to-dark pigment pattern. Weller’s new white scar = outer white border. Figure 5, Figure 6, Figure 7A, Figure 11A, Figure 12A, Figure 13A,B, Figure 14A,B, Figure 18, Figure 19A,B, Figure 22B,C, Figure 23A–D, Figure 24A, Figure 25A,B, Figure 26, Figure 29A,B, Figure 30A, Figure 33, Figure 34, Figure 40 and Figure 41.
Stage 4: Complete wound healing. At 61–476 days/1.3 years, scar pigmentation was normal or nearly normal. The appearance of scars varied from indistinguishable to slightly darker or lighter than the surrounding normal skin. Repigmentation of epidermal tissue varied between individuals, but in no cases did it occur prior to 61 days post-injury. Some scars were indented a few millimeters.	Stage 5: Healed wound without cellular and vascular blubber. Pigmentation similar to surrounding skin, *n* = 13 scars.	Bi-colored light-to-dark pigment pattern. Weller scar appearance varied, comparable to a faded light-to-dark pigment pattern. Figure 35 and Figure 36.

### 3.3. Photo Confirmation of Su et al.’s [6,22,23] Five Healing Stages

Two studies showed the links between macroscopic markers and histology on live and dead free-ranging dolphins, respectively [6,15,22,23]. Extensive comparisons of the Faithful Canvas collection [12], Bruce-Allen and Geraci [15], and Su et al. [6] confirmed strong macroscopic matches (Figure 3, Figure 4, Figure 5, Figure 6, Figure 7, Figure 8, Figure 9, Figure 10, Figure 11, Figure 12, Figure 13, Figure 14, Figure 15, Figure 16, Figure 17, Figure 18, Figure 19, Figure 20, Figure 21, Figure 22, Figure 23, Figure 24, Figure 25, Figure 26, Figure 27, Figure 28, Figure 29, Figure 30, Figure 31, Figure 32, Figure 33, Figure 34, Figure 35, Figure 36, Figure 37, Figure 38, Figure 39, Figure 40 and Figure 41). Matches reveal macroscopic markers of the underlying histology of inflammation, proliferation, and remodeling.

Postmortem ischemia in stranded dolphins reported by Su et al. [6,22,23] froze cellular patterns in place at death, providing valuable snapshots of immune activity. However, the major constraint on Su et al.’s histology data was that the number of healing days was unknown. Thus, Faithful Canvas data [12] expanded Su et al.’s stages by providing estimated timeframes of each Su stage and illustrating dynamic changes therein. Figure 3, Figure 4, Figure 5, Figure 6 and Figure 7 show common macroscopic exemplars that reflect the stages described by Su et al. [6,22,23]. Figure 8, Figure 9, Figure 10, Figure 11, Figure 12, Figure 13, Figure 14, Figure 15, Figure 16, Figure 17, Figure 18, Figure 19, Figure 20, Figure 21, Figure 22, Figure 23, Figure 24, Figure 25, Figure 26, Figure 27, Figure 28, Figure 29, Figure 30, Figure 31, Figure 32, Figure 33, Figure 34, Figure 35, Figure 36, Figure 37, Figure 38, Figure 39, Figure 40 and Figure 41 are excerpts from healing histories [12] to illustrate matches.

Su et al. [6,22,23] described stage 1 wounds as fresh, with sharp edges lined by pale, edematous epidermal cells, minimal bleeding, and intercellular edema in the epidermis. Figure 3A–C illustrate fresh wounds on a live dolphin with sharp edges and edema. Figure 3A shows the poorly apposed edges of degloved wounds that expose underlying tissue. Figure 3B,C show well-apposed edges of surgical slices that do not expose underlying tissue. Figure 8A–C, Figure 15A, Figure 20A, Figure 22A, Figure 27A,B and Figure 37 also show fresh wounds.

Stages 2 and 3 [6,22,23] are early and mature healing wounds, respectively. Su et al. describe stage 2 wounds in the initial healing phase without granulation tissue, characterized by raised edges. Histologically, yellow-white tissue composed of inflammatory cell infiltration, necrotic collagen, and necrotic adipocytes outlined wound edges and covered surfaces, with intra- and intercellular edema from hyperemia and hemorrhage in blubber adjacent to the wound site. Blubber at the wound had been replaced by a loose fiber network of fibrin, erythrocytes, and inflammatory cells. The macroscopic features of Su stage 2 wounds are confirmed by Faithful Canvas [12] wounds in a bi-colored dark-to-light pigment pattern (Figure 4A, Figure 9A,D, Figure 10A, Figure 16A,B, Figure 17, Figure 21B and Figure 38).

Su et al. [6,22,23] described a stage 3 wound as a mature, open-healing wound with swollen, puckered wound edges. Histologically, visible granulation was composed of fibrocytes, fibroblasts, and thin collagen III fibers. Angiogenesis was ongoing. Melanocytes, melanin, and the dermal–epidermal junction were visible in developing epithelial tongues of keratinocytes in the neoepidermis. Blubber adjacent to the wound was still replaced by cells and fibers. Figure 4A illustrates the bi-colored dark-to-light pigment pattern on a dorsal fin insertion slice from five months of chronic fishing line entanglement, during which it exhibited the dark-to-light pigment pattern continuously. Its thick upper ‘lip’ is typical of dorsal fin leading edge slice wounds. Only after stranding personnel removed the fishing line and healing began did the wound switch to the light-to-dark pigment pattern (Figure 5A), which matches a Su stage 4 wound. The macroscopic features of Su stage 3 wounds are confirmed by Faithful Canvas [12] wounds in a tri-colored dark-to-light pigment pattern (Figure 4A, Figure 9B,C, Figure 10B,C, Figure 21A,C,D, Figure 22D, Figure 28A,B, Figure 31, Figure 32 and Figure 39).

**Figure 6:**
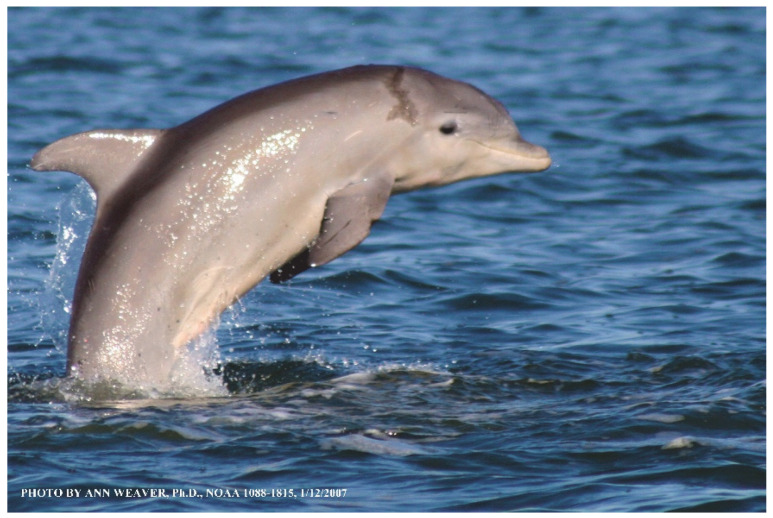
Example of long-term hyperpigmented head scar. Source: [12] Vol. 3 Head Bites, Ch. 6 Keynote Case, Scarface, Photo 8. Scarface’s estimated age: 619 days/1.7 years old; 12 January 2007 (60 °F/16 °C).

**Figure 7:**
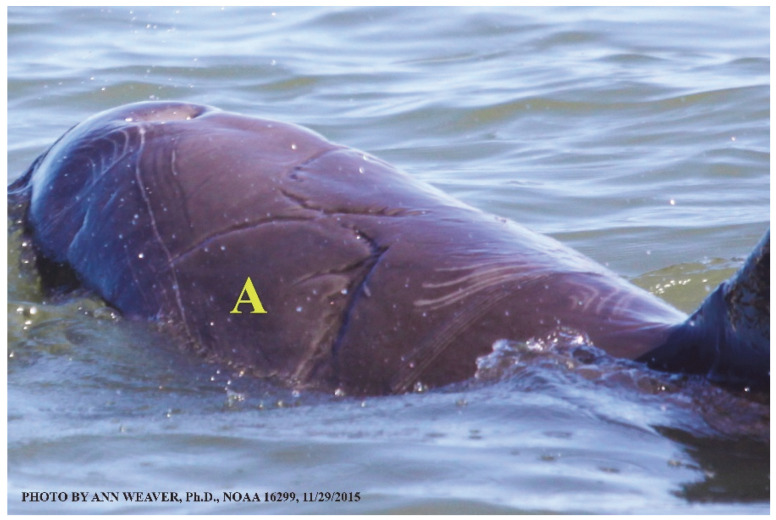
A: Enduring atrophic scars. Source: [12] Vol. 3 Head Bites, Ch. 10 Point. Photo 5. Adult male, no age estimate; 29 November 2015 (67 °F/19 °C).

Stages 4 and 5 are healed scars characterized by gradual restoration of normal skin architecture. Su et al. described stage 4 scars as immature healed wounds that showed complete closure but incomplete repigmentation. Histologically, Su et al.’s stage 4 wound surfaces were fully epithelialized, without cellular edema, and showed new thin collagen fibers in the dermis and blubber, oriented parallel to the skin surface (lacking the basketweave of the original collagen), numerous blood vessels, and a restored dermal–epidermal junction, indicating a well-structured neoepidermis. Adipose tissues were limited to cellular and vascular blubber. Melanin granules were present in all layers of the neoepidermis. Nerve fibers were rarely observed. The macroscopic features of Su stage 4 wounds are confirmed by Faithful Canvas [12] scars in the light-to-dark pigment pattern, which qualify by incomplete repigmentation, but also show incomplete filling (Figure 6, Figure 7A, Figure 11A, Figure 12A, Figure 13A,B, Figure 14A,B, Figure 18, Figure 19A,B, Figure 22B,C, Figure 23A–D, Figure 24A, Figure 25A,B, Figure 26, Figure 29A,B, Figure 30A, Figure 33, Figure 34, Figure 40 and Figure 41). Figure 6 shows incomplete repigmentation of the scar from a shark bite in the head of a neonatal dolphin, which healed to a jagged hypopigmented scar by 9 months and remained hyperpigmented for 14 years before it matched the now-adult dolphin’s background color. Figure 6 also shows the stretching and flexing that dolphin scar tissue must withstand.

Faithful Canvas scars [12] in Su et al.’s stage 4 [6,22,23] repigment eventually but remain atrophic. Examples include the atrophic scars in Figure 7, which were visible from 2007 to 2024 (end of study). The caudal leg of the adult male Point’s upside-down asymmetric crescent scar is more atrophic than its cranial leg, one of countless examples of scar asymmetry. Its inside scar edge is also heavily irregular, suggesting poorly apposed wound edges from wrenching and slicing, whereas its outside scar edge is smooth. Atrophic scars do not meet the Su et al. criterion of full tissue restoration macroscopically.

Su et al. [6,22,23] described stage 5 as mature, healed, closed wounds with few or no contraction lines, evenly pigmented, with surrounding unwounded skin. The amount of adipose tissue varied among different samples, but histologically, both dermis and blubber remained less cellular and vascular (a description reminiscent of atrophic scars). Few nerve fibers were observed in the dermis. Most Stage 5 wounds still had thin collagen III fibers, with only a few containing thick collagen I fibers. The macroscopic features of Su et al.’s stage 5 wounds are tentatively confirmed by the few Faithful Canvas [12] scars that filled in and repigmented to the background color (Figure 35 and Figure 36).

**Figure 8:**
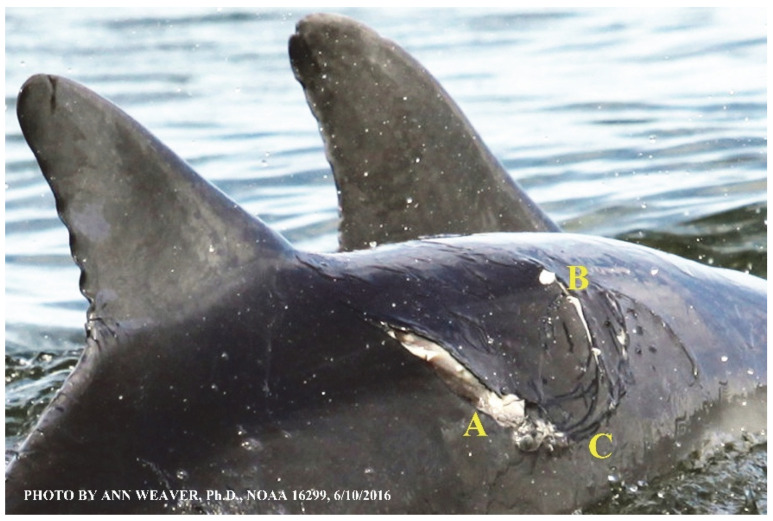
Day 1. Su et al. stage 1: fresh 20 min-old wound with sharp shoulders (C), then local swelling within 30 min creating indistinct outer halos, no blood, some bulging blubber (A,B). Source: [12] Vol. 2 Body Bites, Ch. 2 Keynote Case, Saga, Photo 10. Saga’s estimated age: 399 days/1.1 years old; 10 June 2016 (83 °F/28 °C). 9:46 a.m.

**Figure 9:**
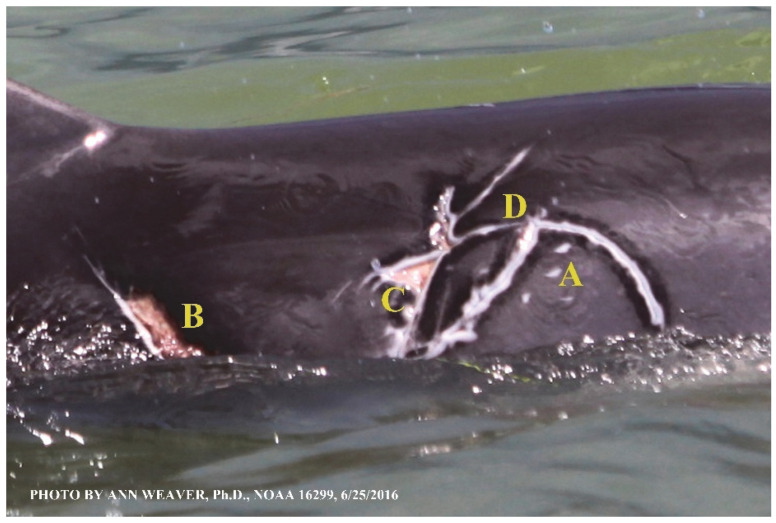
Day 15/2.1 weeks. Su et al. stage 2: A,D: bi-colored dark-to-light pigment pattern. Su et al. stage 3: B,C: tri-colored dark-to-light pigment pattern. Source: [12] Vol. 2 Body Bites, Ch. 2 Keynote Case, Saga, Photo 13. Saga’s estimated age: 414 days/1.1 years old; 25 June 2016 (83 °F/28 °C).

**Figure 10:**
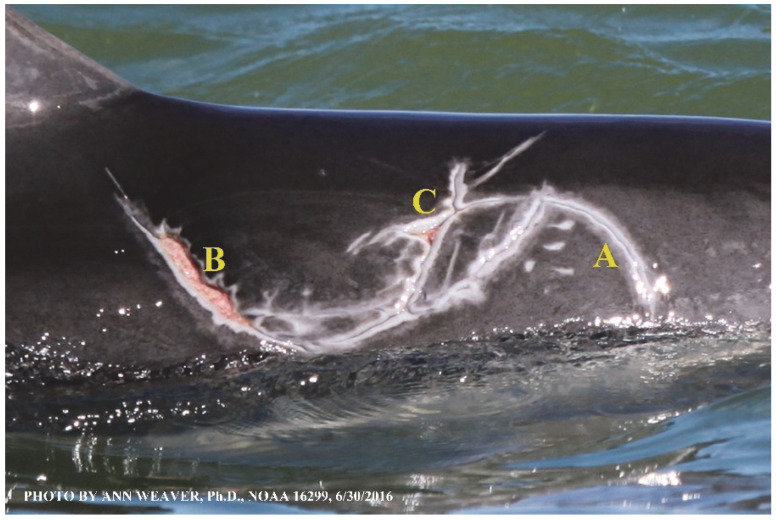
Day 20/2.9 weeks. Dynamic changes in dark-to-light pigment patterns took place in the next week. Su et al. stage 2: A: bi-colored dark-to-light pigment pattern. Su et al. stage 3: B,C: tri-colored dark-to-light pigment pattern. Source: [12] Vol. 2 Body Bites, Ch. 2 Keynote Case, Saga, Photo 16. Saga’s estimated age: 419 days/1.1 years old; 30 June 2016 (83 °F/28 °C).

**Figure 11:**
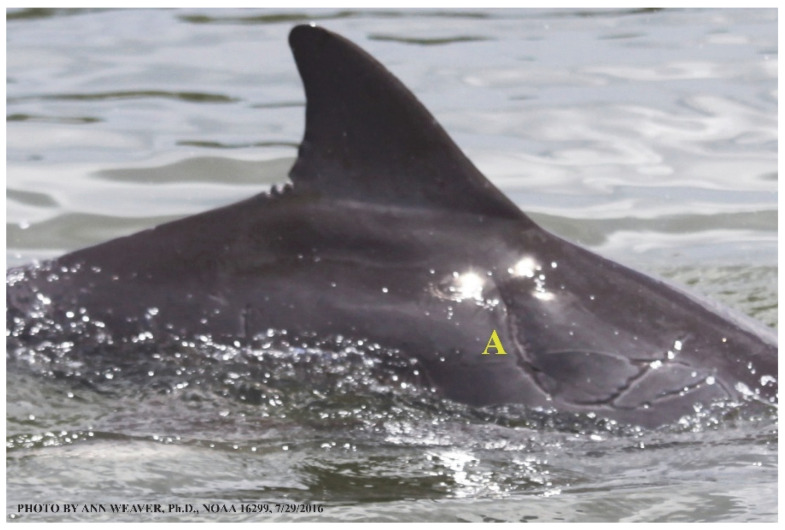
A: Day 49/7.0 weeks. Su et al. stage 4: light-to-dark pigment pattern with preliminary seals and unpigmented spots by 7 weeks. Source: [12] Vol. 2 Body Bites, Ch. 2 Keynote Case, Saga, Photo 18. Saga’s estimated age: 448 days/1.2 years old; 29 July 2016 (84 °F/29 °C).

**Figure 12:**
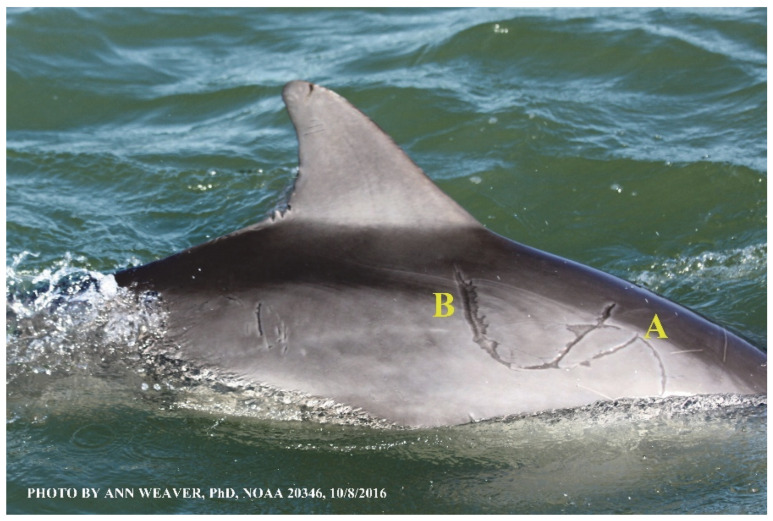
Day 120/4.0 months. Su et al.’s stage 4 characteristics of incomplete repigmentation (A,B) and atrophism (B) persisted for 4 months. Source: [12] Vol. 2 Body Bites, Ch. 2 Keynote Case, Saga, Photo 26. Saga’s estimated age: 519 days/1.4 years old; 8 October 2016 (76 °F/24 °C).

**Figure 13:**
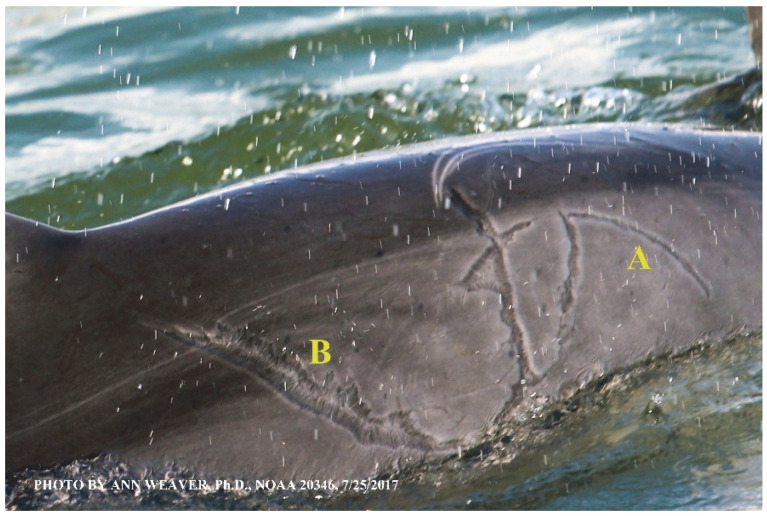
A: Day 410/1.1 years. Su et al. stage 4: hyperpigmentation of surgical scar. B: interiors of degloved wounds develop visible striated tissue escarpments and thick hypopigmented riverine scars. Stage 4 characteristics of incomplete repigmentation and atrophism remained across the first year. Source: [12] Vol. 2 Body Bites, Ch. 2 Keynote Case, Saga, Photo 30. Saga’s estimated age: 809 days/2.2 years old; 25 July 2017 (84 °F/29 °C).

**Figure 14:**
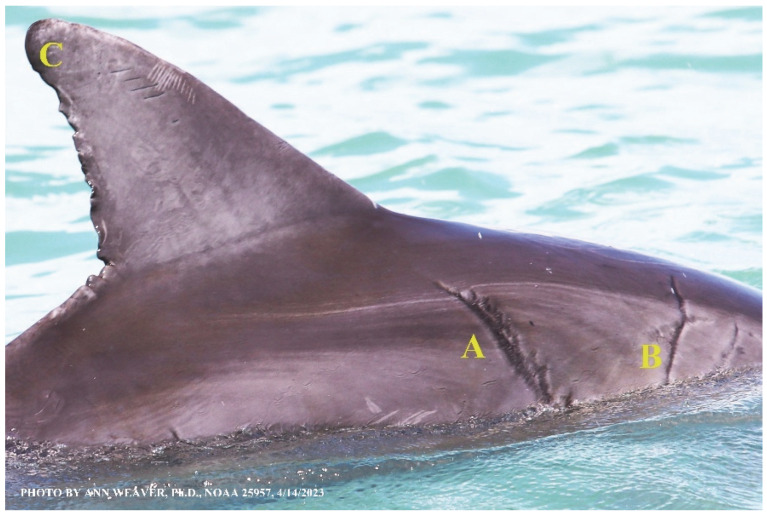
Day 2499/6.8 years. Su et al.’s stage 4 characteristics of degree of repigmentation and atrophism (A,B) remained across the next 6.8 years (end of study). On the top of the right face of Saga’s dorsal fin (C), the slit that was visible for 5.6 years from 2016 through 2021 became obliterated during the summer of 2022 and was no longer visible. Source: [12] Vol. 2 Body Bites, Ch. 2 Keynote Case, Saga, Photo 35. Saga’s estimated age: 2989 days/7.9 years old; 14 April 2023 (72 °F/22 °C).

**Figure 15:**
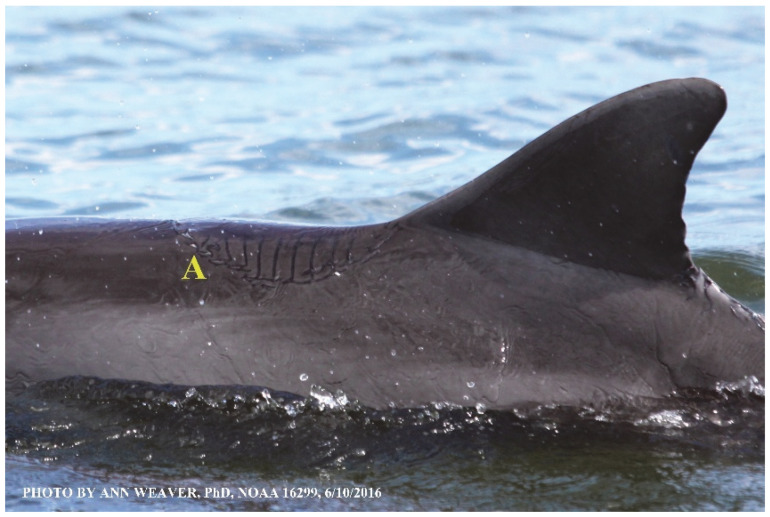
Day 1. Su et al. stage 1: 10 min-old fresh wound from smooth parallel shark tooth rake drag marks (A): still dark, hard to see, sharp shoulders, no blood, no blubber bulges, no pigment pattern. Source: [12] Vol. 2 Body Bites, Ch. 2 Keynote Case, Saga, Photo 39. The day the shark bit Saga. Saga’s estimated age: 399 days/1.1 years old. 10 June 2016 (83 °F/28 °C).

**Figure 16:**
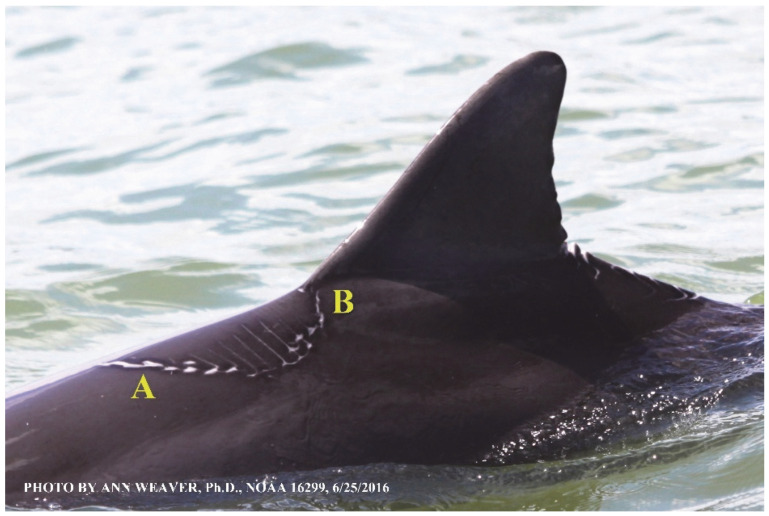
Day 15/2.1 weeks. Su et al. stage 2: wound shifted to the bi-colored dark-to-light pigment pattern through 2.1 weeks. Pigment differences in white border development depend on wound depth, with wider white borders developing around deeper portions of wounds (A) and thin dark borders on shallower wounds (B). Source: [12] Vol. 2 Body Bites, Ch. 2 Keynote Case, Saga, Photo 40. Saga’s estimated age: 414 days/1.1 years old; 25 June 2016 (83 °F/28 °C).

**Figure 17:**
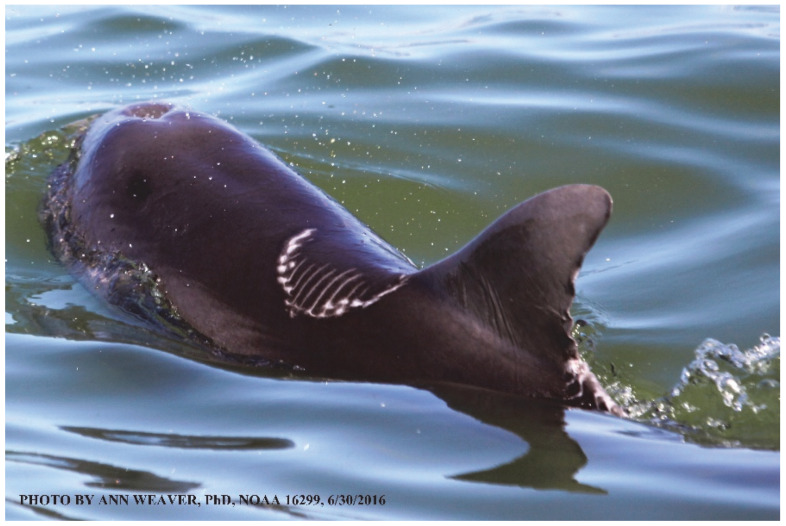
Day 20/2.9 weeks. Su et al. stage 2: wound showed dynamic change in which the inner white border ‘brightened’ (lightening and sometimes thickening) by 2.9 weeks. Source: [12] Vol. 2 Body Bites, Ch. 2 Keynote Case, Saga, Photo 42. Saga’s estimated age: 419 days/1.1 years old; 30 June 2016 (83 °F/28 °C).

**Figure 18:**
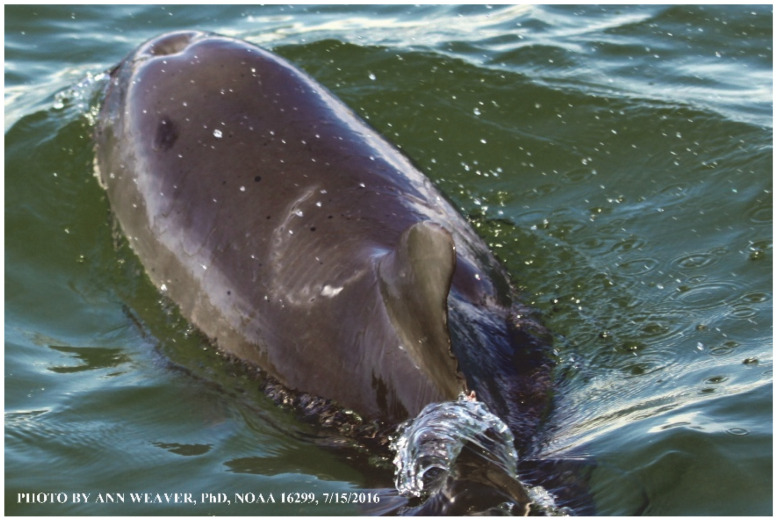
Day 35/5.0 weeks. Su et al. stage 4: shifted to the light-to-dark pigment pattern by 5 weeks. Source: [12] Vol. 2 Body Bites, Ch. 2 Keynote Case, Saga, Photo 43. Saga’s estimated age: 434 days/1.2 years old; 15 July 2016 (84 °F/29 °C).

**Figure 19:**
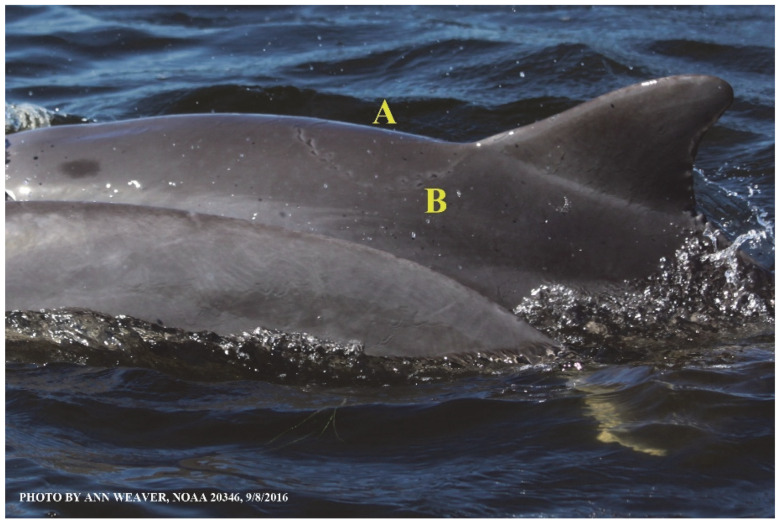
Day 90/3.0 months. A: Scars from the bite of the shark’s lower jaw mostly blended in (A) although Su et al. stage 4 scar characteristics remained (B: continuing atrophism) with some changes (slow repigmentation) across the next 6.8 years (end of study). Source: [12] Vol. 2 Body Bites, Ch. 2 Keynote Case, Saga, Photo 46. Saga’s estimated age: 489 days/1.3 years old; 8 September 2016 (82 °F/28 °C).

**Figure 20:**
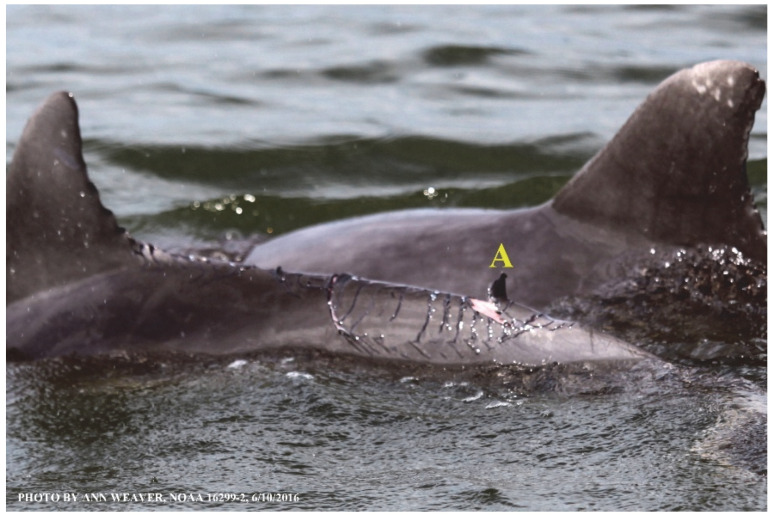
A: Day 1. Su et al. stage 1: fresh 9 min-old peduncle ridge shark bites of varying severity with sharp shoulders, no blood. Source: [12] Vol. 2 Body Bites, Ch. 2 Keynote Case, Saga, Photo 57. The day the shark bit Saga. Saga’s estimated age: 399 days/1.1 years old; 10 June 2016 (83 °F/28 °C).

**Figure 21:**
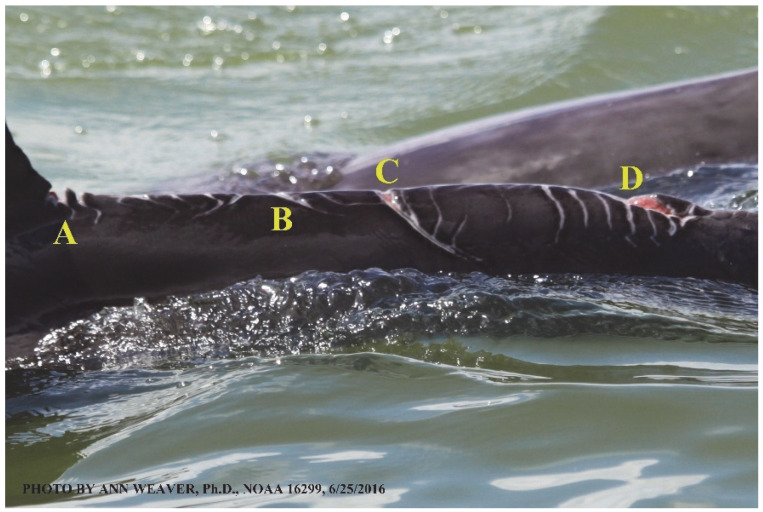
Day 15/2.1 weeks. Su et al. stage 2: A,B: tooth rakes. Su et al. stage 3: C,D shows minor degloving, revealing underlying granulation. Source: [12] Vol. 2 Body Bites, Ch. 2 Keynote Case, Saga, Photo 61. Saga’s estimated age: 414 days/1.1 years old; 25 June 2016 (83 °F/28 °C).

**Figure 22:**
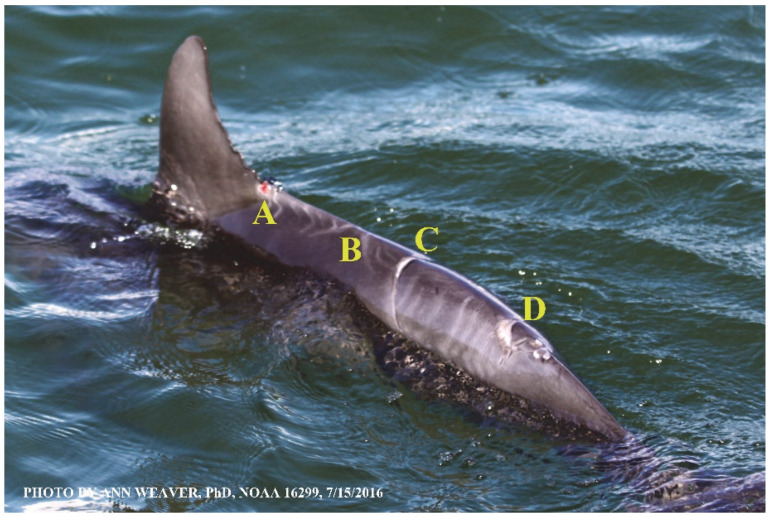
Day 35/5.0 weeks. Su et al. stage 1: A shows a fresh wound. Su et al. stage 4: B. Su et al. stage 4: C shows a preliminary seal. Su et al. stage 3: D shows a slit of granulation. Wounds exhibited dynamic changes through 5 weeks. Source: [12] Vol. 2 Body Bites, Ch. 2 Keynote Case, Saga, Photo 67. Saga’s estimated age: 434 days/1.2 years old; 15 July 2016 (84 °F/29 °C).

**Figure 23:**
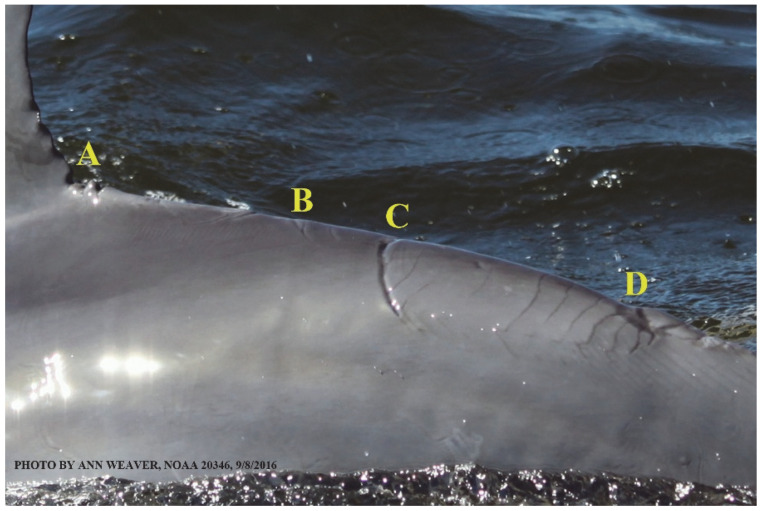
Day 90/3.0 months. Su et al. stage 4: A–D shows a light-to-dark pigment pattern. Wounds of differing severity developed varying degrees of pigmentation. D shows conspicuous hyperpigmentation (for three months). Scars from shark drag marks inside crescent shark bite wounds retained the original jagged or curvilinear nature of wounds. Source: [12] Vol. 2 Body Bites, Ch. 2 Keynote Case, Saga, Photo 70. Saga’s estimated age: 489 days/1.3 years old; 8 September 2016 (82 °F/28 °C).

**Figure 24:**
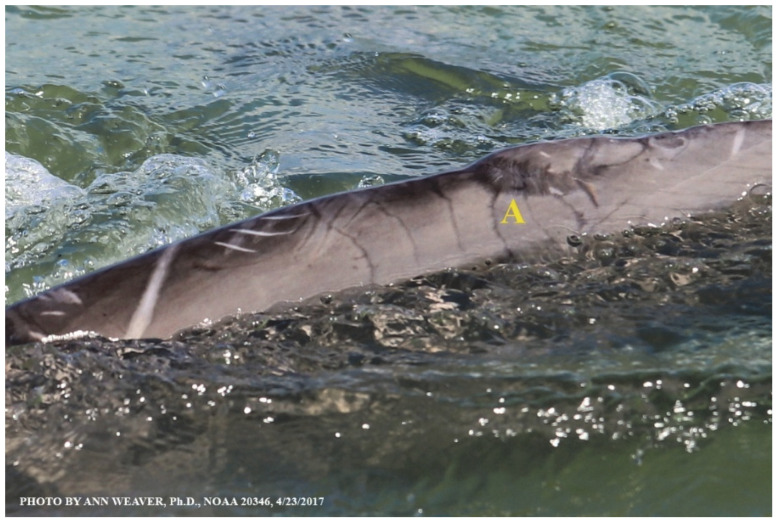
Day 317/10.6 months. Su et al. stage 4: A shows conspicuous hyperpigmentation and developing striations through 10+ months. Source: [12] Vol. 2 Body Bites, Ch. 2 Keynote Case, Saga, Photo 76. Saga’s estimated age: 716 days/2 years old; 23 April 2017 (72 °F/22 °C).

**Figure 25:**
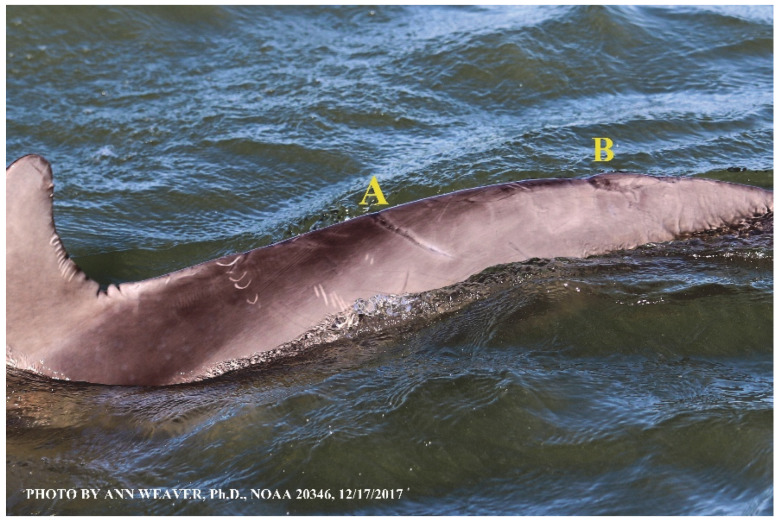
Day 555/1.5 years. Su et al.’s stage 4: A,B show conspicuous hyperpigmentation and striations through 1.5 years. Source: [12] Vol. 2 Body Bites, Ch. 2 Keynote Case, Saga, Photo 82. Saga’s estimated age: 954 days/2.6 years old; 17 December 2017 (63 °F/17 °C).

**Figure 26:**
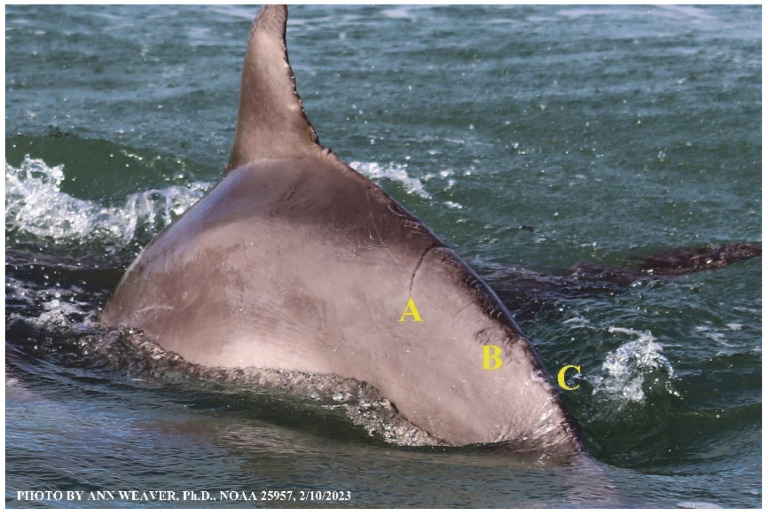
Day 2436/6.7 years. Su et al.’s stage 4: Scars A, B, & C show that they were still hyperpigmented and atrophic over 6 years later. Source: [12] Vol. 2 Body Bites, Ch. 2 Keynote Case, Saga, Photo 87. Saga’s estimated age: 2835 days/7.8 years old; 10 February 2023 (61 °F/16 °C).

**Figure 27:**
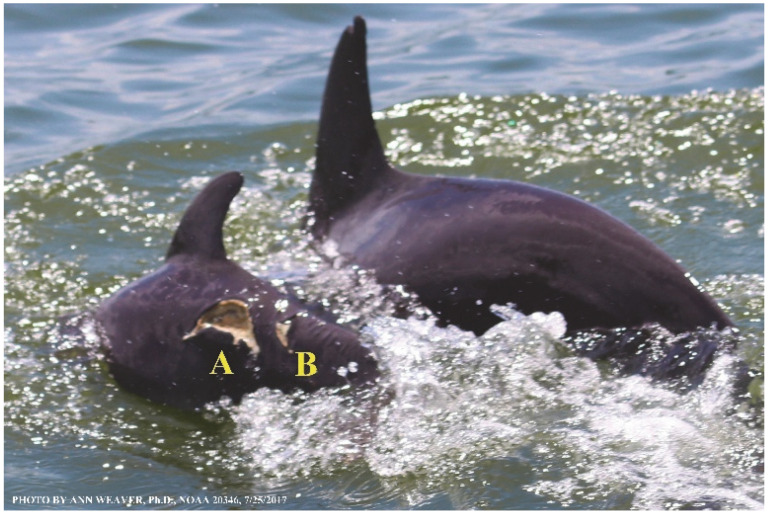
Day 1. Su et al. stage 1: fresh degloved shark bite wounds (A), and bites of lesser severity (B), crescent apex edges already swollen and slightly retracted, no blood, and exposed blubber shows the yellow-beige color of fresh bottlenose dolphin wounds. Source: [12] Vol. 9 Peduncle Bites, Ch. 2 Keynote Case, Pinky, Photo 4. Pinky’s estimated age: 54 days/7.7 weeks old; 25 July 2017 (84 °F/29 °C).

**Figure 28:**
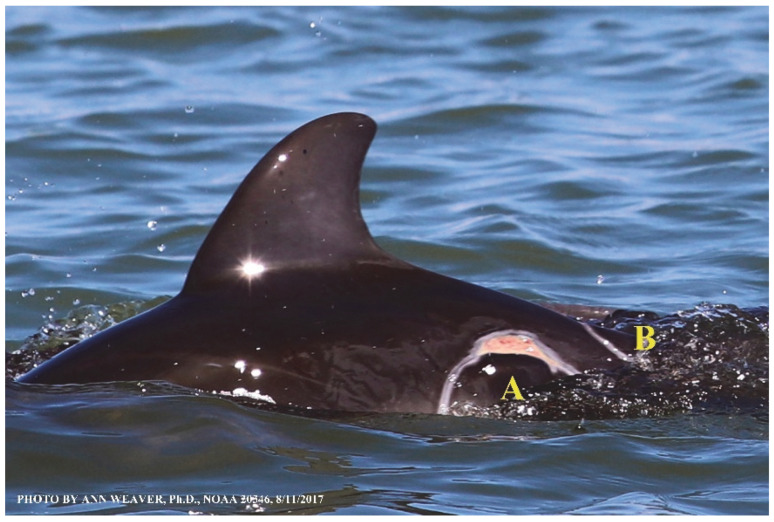
Day 17/2.4 weeks. Su et al. stage 3: wounds developed a tri-colored dark-to-light pigment pattern with exposed granulation (A), partial closure (B), and a potential example of maritime tertiary intention healing through 2.4 weeks. Source: [12] Vol. 9 Peduncle Bites, Ch. 2 Keynote Case, Pinky, Photo 7. Pinky’s estimated age: 71 days/2.4 months old; 11 August 2017 (85 °F/29 °C).

**Figure 29:**
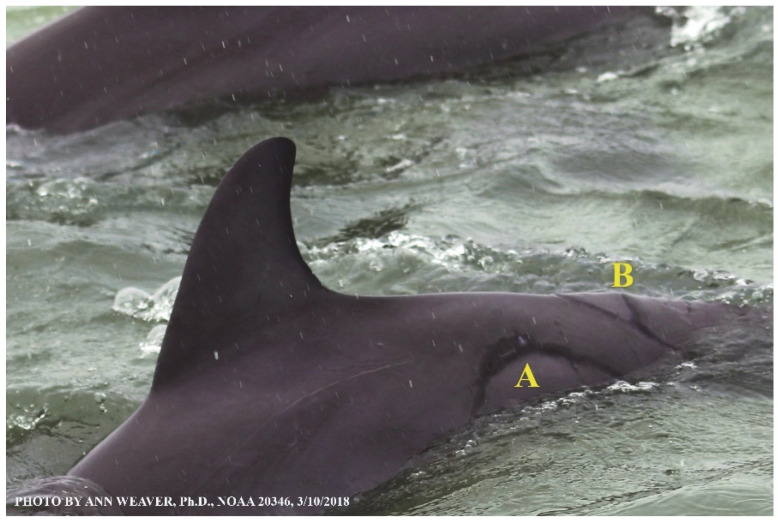
Day 228/7.6 months. Su et al. stage 4: new scars shifted to light-to-dark pigment pattern with obvious hyperpigmentation for 7.6 months (A,B). The best-apposed wound edges on the crescent legs contracted first, forming an initial seal. The least-apposed edges of the gnarly apex gouge that exposed pink underlying tissue (Figure 28A above) healed to the gnarliest scar (Figure 30B below). Source: [12] Vol. 9 Peduncle Bites, Ch. 2 Keynote Case, Pinky, Photo 19. Pinky’s estimated age: 282 days/9.4 months old; 10 March 2018 (67 °F/19 °C).

**Figure 30:**
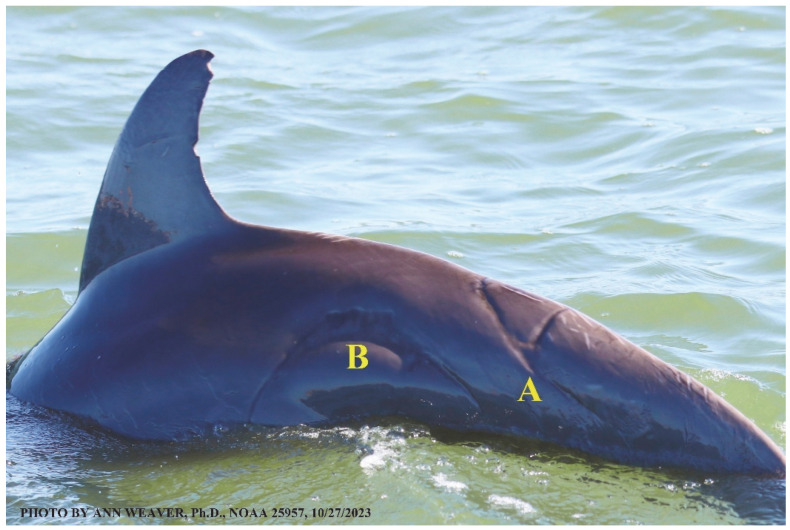
Day 2285/6.3 years. Su et al. stage 4: A,B show that the characteristics of slow repigmentation and persistent atrophism persisted for the next 6+ years (end of study). Source: [12] Vol. 9 Peduncle Bites, Ch. 2 Keynote Case, Pinky, Photo 42. Pinky’s estimated age: 2339 days/6.4 years old; 27 October 2023 (76 °F/24 °C).

**Figure 31:**
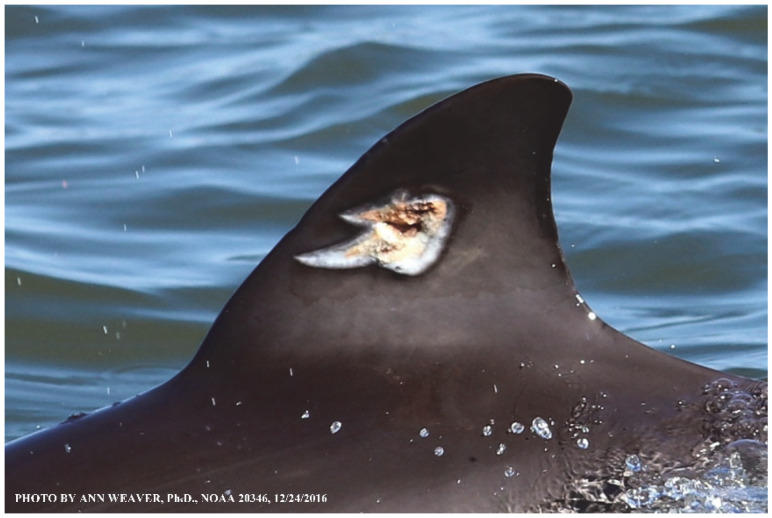
Day 14/2.0 weeks. Su et al. stages 2 and 3: semi-fresh gouge wound of the dorsal fin, partial development of tri-colored dark-to-light pigment pattern on top half of gouge. Source: [12] Vol. 6 Dorsal Fin Gouges, Ch. 2 Keynote Case, Stem, Photo 3. Stem’s estimated age: 145 days/4.8 months old; 24 December 2016 (63 °F/17 °C).

**Figure 32:**
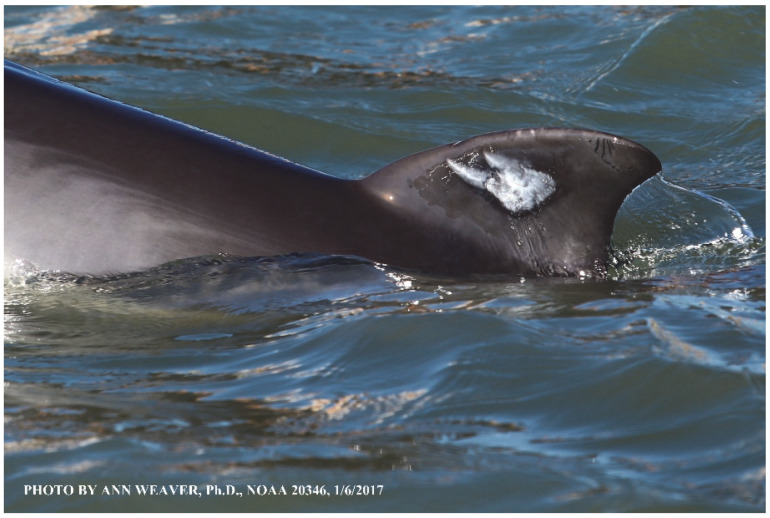
Day 27/3.9 weeks. Su et al. stage 3: wound face fully covered with textured inner white border approximately a month later. Source: [12] Vol. 6 Dorsal Fin Gouges, Ch. 2 Keynote Case, Stem, Photo 6. Stem’s estimated age: 158 days/5.3 months old; 6 January 2017 (60 °F/16 °C).

**Figure 33:**
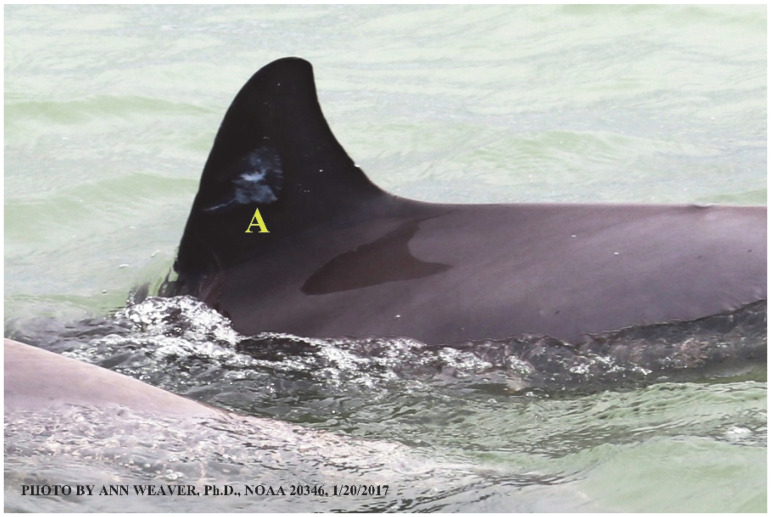
Day 41/5.9 weeks. Su et al. stage 4: new scar shifted to light-to-dark pigment pattern with unpigmented preliminary seal by approximately 6 weeks (A). Source: [12] Vol. 6 Dorsal Fin Gouges, Ch. 2 Keynote Case, Stem, Photo 7. Stem’s estimated age: 172 days/5.7 months old; 20 January 2017 (60 °F/16 °C).

**Figure 34:**
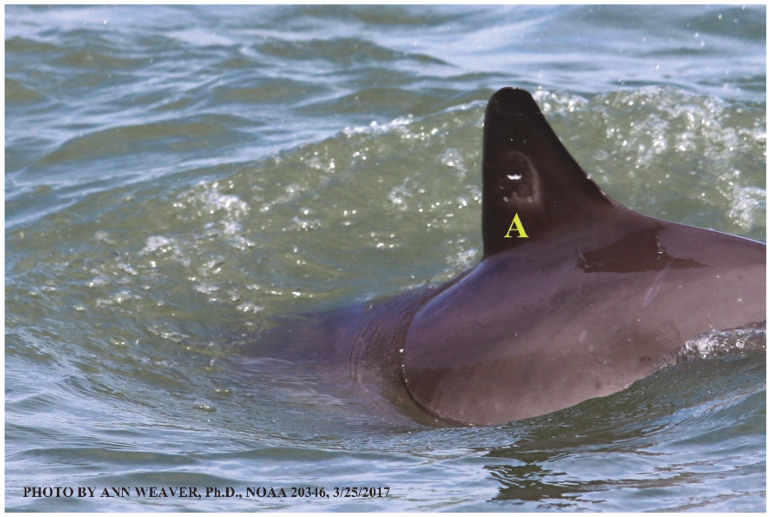
Day 105/3.5 months. Su et al.’s stage 4: tri-colored light-to-dark pigment pattern of the scar remained stable for 3.5 months (A). Source: [12] Vol. 6 Dorsal Fin Gouges, Ch. 2 Keynote Case, Stem, Photo 9. Stem’s estimated age: 236 days/7.9 months old; 25 March 2017 (67 °F/19 °C).

**Figure 35:**
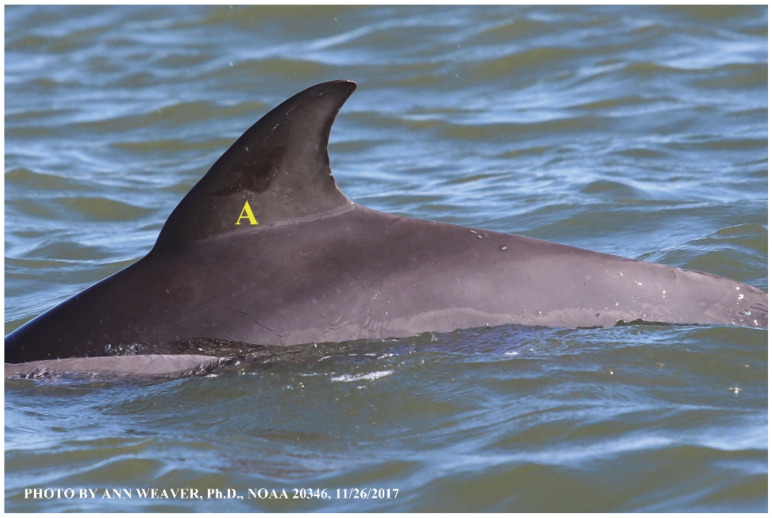
Day 351/11.7 months. Su et al.’s stage 5: scar remained hyperpigmented but slowly repigmented across the next 5 years until Stem’s death in May 2022 (A). This is one of the few Faithful Canvas healing histories that meet Su et al.’s [6,22,23] stage 5 criteria. Source: [12] Vol. 6 Dorsal Fin Gouges, Ch. 2 Keynote Case, Stem, Photo 12. Stem’s estimated age: 482 days/1.3 years old; 26 November 2017 (67 °F/19 °C).

**Figure 36:**
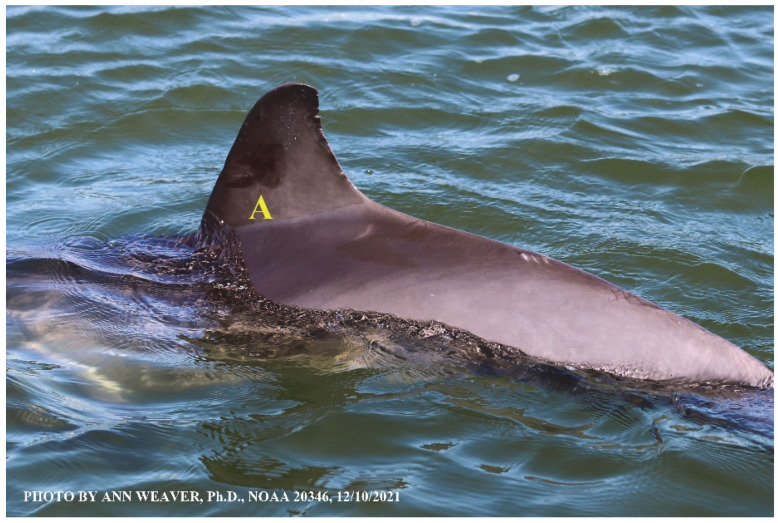
Day 1826/5.0 years. Su et al.’s stage 5: scar remained hyperpigmented but filled in across the next 5 years until Stem’s death in May 2022 (A). One of the few cases in the Faithful Canvas collection [12] that meets Su et al.’s [6,22,23] stage 5 criteria. Source: [12] Vol. 6 Dorsal Fin Gouges, Ch. 2 Keynote Case, Stem, Photo 15. Stem’s estimated age: 1957 days/5.4 years old; 10 December 2021 (63 °F/17 °C).

**Figure 37:**
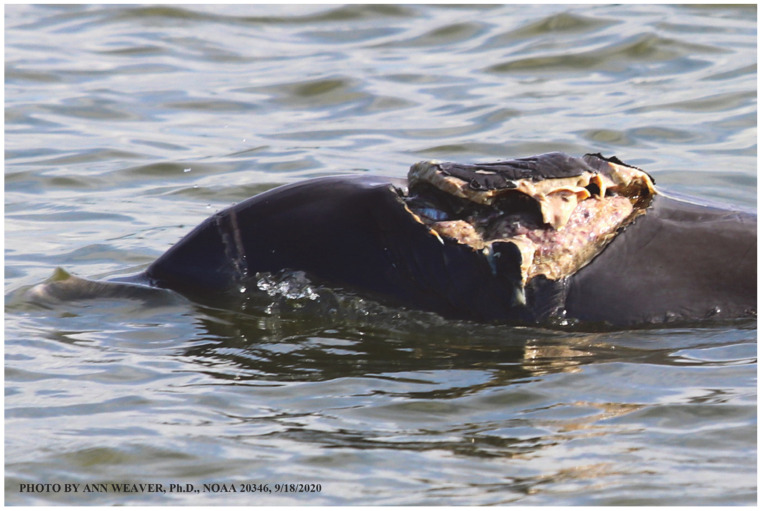
Day 1. Su et al. stage 1: fresh, brutal, degloved wound from a shark bite over the top of the right side of the head of an adult female P. Source: [12] Vol. 3 Head Bites, Ch. 3 Keynote Case, P, Photo 5. The day she was bitten by the shark was 18 September 2020 (82 °F/28 °C).

**Figure 38:**
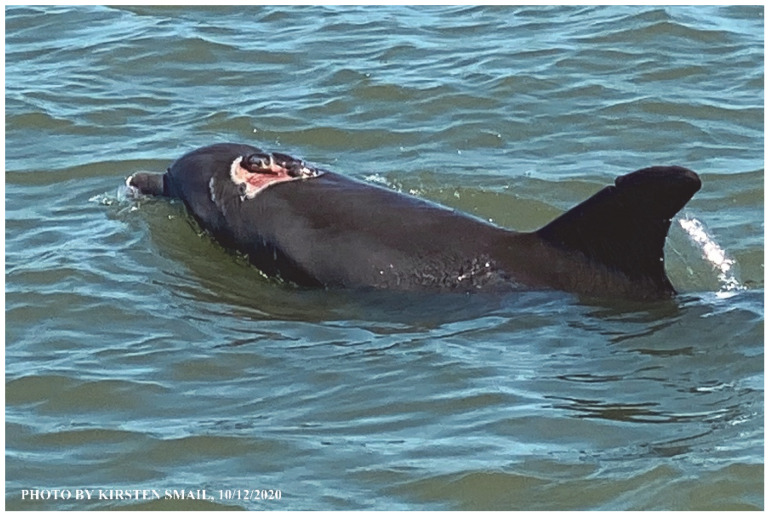
Day 24/3.4 weeks. Su et al. stage 3: brutal degloved wound from a shark bite on the head of an adult female P, as of 3+ weeks of healing. The white border is easy to see. Source: [12] Vol. 3 Head Bites, Ch. 3 Keynote Case, P, Photo 10; 12 October 2020 (76 °F/24 °C).

**Figure 39:**
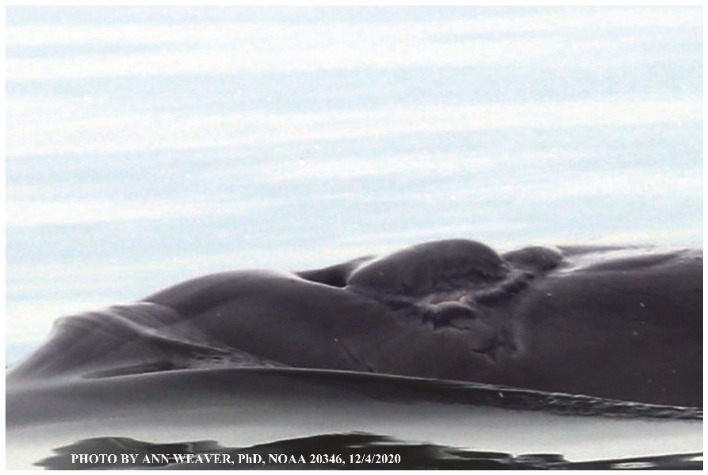
Day 77/2.6 months. Su et al. stage 3: brutal degloved wound from a shark bite on the head of an adult female P was still partially open as of 77 days/2.6 months of healing. This shark bite finally closed after 2.8 months of healing. Source: [12] Vol. 3 Head Bites, Ch. 3 Keynote Case, P, Photo 12; 4 December 2020 (63 °F/17 °C).

**Figure 40:**
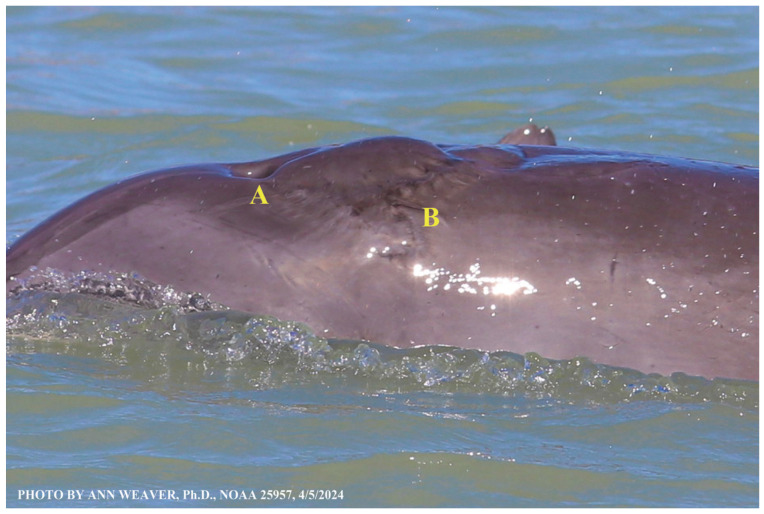
Day 1295/3.5 years. Left view of a bottlenose dolphin’s head and shark bite scars. A: points to the blowhole for orientation. B: plait-like scar tissue. Su et al.’s stage 4: after 3.5 years of healing and remodeling, scars of the brutal shark bite on the head of adult female P showed signs of smoothing (B) but made it hard or impossible for her to bend her head downward. Source: [12] Vol. 3 Head Bites, Ch. 3 Keynote Case, P, Photo 39A; 5 April 2024 (72 °F/22 °C).

**Figure 41:**
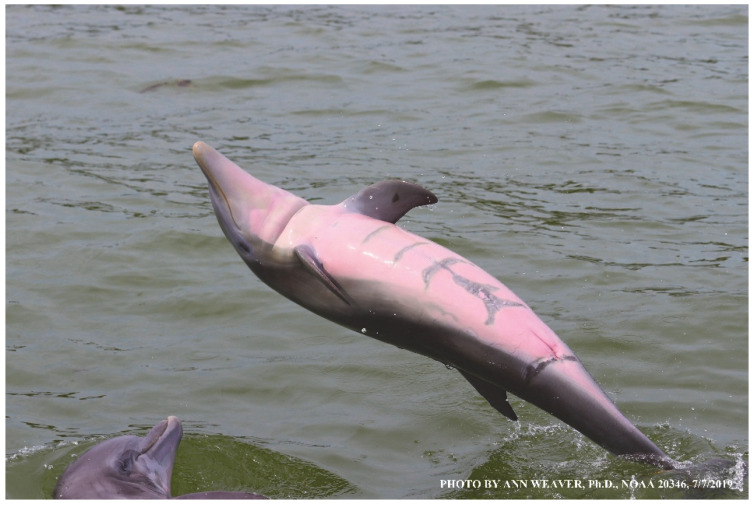
Su et al. stage 4. Scars from propeller strikes on the ventrum of a yearling bottlenose dolphin, Sahara, healed to gray, even though dolphin ventrums are white from countershading. Her ventrum is temporarily pink due to sustained socializing. Source: [12] Vol. 10 Propeller Strikes, Ch. 3, Sahara.

Figure 8, Figure 9, Figure 10, Figure 11, Figure 12, Figure 13, Figure 14, Figure 15, Figure 16, Figure 17, Figure 18, Figure 19, Figure 20, Figure 21, Figure 22, Figure 23, Figure 24, Figure 25, Figure 26, Figure 27, Figure 28, Figure 29, Figure 30, Figure 31, Figure 32, Figure 33, Figure 34, Figure 35, Figure 36, Figure 37, Figure 38, Figure 39 and Figure 40 illustrate dynamic changes in Faithful Canvas [12] macroscopic markers of healing as six photo clusters, each showing sequential changes in the same wound scar over time (top shark jaw creating dorsum bite: Figure 8, Figure 9, Figure 10, Figure 11, Figure 12, Figure 13 and Figure 14; bottom shark jaw creating dorsum bite: Figure 15, Figure 16, Figure 17, Figure 18 and Figure 19; shark bites along peduncle ridge: Figure 20, Figure 21, Figure 22, Figure 23, Figure 24, Figure 25 and Figure 26; shark bites on peduncle: Figure 27, Figure 28, Figure 29 and Figure 30; gouge wound on dorsal fin: Figure 31, Figure 32, Figure 33, Figure 34, Figure 35 and Figure 36, brutal shark bite on head: Figure 37 and Figure 4). Propeller strikes on ventrum are illustrated on Figure 41. The first three clusters (Figure 8, Figure 9, Figure 10, Figure 11, Figure 12, Figure 13, Figure 14, Figure 15, Figure 16, Figure 17, Figure 18, Figure 19, Figure 20, Figure 21, Figure 22, Figure 23, Figure 24, Figure 25 and Figure 26) illustrate healing of different shark bites from our eye-witnessed episode of a shark biting a yearling 11 times; exact days of healing are known. One dynamic aspect of the initial dark-to-light pigment pattern seen across photo clusters is the correspondence between wound severity and pigment pattern duration and vibrancy (conspicuousness): least vibrant in superficial stage 2 wounds (Figure 21A,B), moderate in partial-thickness wounds (Figure 31), and most vibrant in full-thickness wounds (Figure 4A, Figure 9B,C, Figure 10B,C, Figure 21C,D and Figure 28A,B). Well-apposed edges often develop fainter pigment borders (Figure 10A). Less-apposed edges often develop more conspicuous borders (Figure 10B).

#### 3.3.1. Faithful Canvas [12] Healing History Excerpts of Right Dorsum Shark Bite: Saga

Figure 8, Figure 9, Figure 10, Figure 11, Figure 12, Figure 13 and Figure 14 show the dynamic changes in a shark’s upper jaw bite on the right side of the dorsum on a yearling male bottlenose dolphin named Saga. The author was present when the shark bit the dolphin.

#### 3.3.2. Faithful Canvas [12] Healing History Excerpts from Shark Bite on Left Side of Dorsum: Saga

Figure 15, Figure 16, Figure 17, Figure 18 and Figure 19 show the dynamic changes in a shark’s lower jaw bite on the left side of the dorsum on a yearling male bottlenose dolphin named Saga. This is the other side of the bite shown in Figure 8, Figure 9, Figure 10, Figure 11, Figure 12, Figure 13 and Figure 14.

#### 3.3.3. Faithful Canvas [12] Healing History Excerpts from Peduncle Ridge Shark Bites: Saga

Figure 20, Figure 21, Figure 22, Figure 23, Figure 24, Figure 25 and Figure 26 show the dynamic changes in three peduncle ridge shark bites on a yearling male bottlenose dolphin named Saga.

#### 3.3.4. Faithful Canvas [12] Healing History Excerpts of Left Peduncle Shark Bites: Pinky

Figure 27, Figure 28, Figure 29 and Figure 30 show the dynamic changes in left peduncle shark bites in a neonate named Pinky at 7.7 weeks old.

#### 3.3.5. Faithful Canvas [12] Healing History Excerpts of Gouge out of Left Face of Dorsal Fin: Stem

Figure 31, Figure 32, Figure 33, Figure 34, Figure 35 and Figure 36 show the dynamic changes in a substantial gouge wound out of the left face of the dorsal fin of a 4.8-month-old calf named Stem. His gouge was up to 3 weeks old when we first saw it. Its source is unknown.

#### 3.3.6. Faithful Canvas [12] Healing History Excerpts of Brutal Degloving Shark Bite on Head: P

Figure 37, Figure 38, Figure 39 and Figure 40 show the dynamic changes in a brutal shark bite to the head directly behind the blowhole of an adult female named P. She survived her extensive wound against all odds. We saw her twice on the same survey, in the morning without the bite and in the afternoon with the fresh bite, so the days of healing and remodeling are known. This wound took 2.8 months to seal, the longest in the Faithful Canvas collection.

## 4. Discussion

### 4.1. Accentuation Hypothesis

Despite advances, cetacean immune operations at sea are poorly understood [102,198,199,200]. The evidence presented here tentatively suggests that aspects of bottlenose dolphin healing are accentuated versions of terrestrial mammal healing, resulting in prolonged inflammation, accelerated epidermal proliferation, and exaggerated scarring despite prolonged remodeling. Compared to terrestrial mammals, major differences in dolphin healing stem from thick skin and exaggerated folds of the dermal–epidermal junction (Figure 1); both fully regenerate early in healing, indicating the pivotal importance of their germinative functions [6,15,22,23]. In contrast, the much smoother dermal–epidermal junction in Lanyu pigs is not replaced in full-thickness scars, and only incompletely in partial-thickness scars [117], and loose-skinned lab animals (rodents, rabbits) have a smooth dermal–epidermal junction [97]. In dolphins, strongly localized inflammatory responses may also contribute to enhanced healing abilities [6,15]. Other examples of accentuation include elaborate mechanisms that stop bleeding yet also accommodate prolonged dives without fatal clotting and create longer coagulation times [51], unusually heavy vascularization of blubber [47], and adipose stem cells and larger platelets containing large alpha granules providing ample sources of multiple growth factors that magnify inflammation and proliferation [46]. Cetacean lipokeratinocytes have 3–5 times the volume of human keratinocytes [116]. Finally, dolphin wound healing is unique for the early appearance of melanocytes and melanin during proliferation [15], which augments inflammation [122]; early restoration again attests to their importance in healing and suggests regenerative rather than reparative healing [6]. This intriguing accentuation hypothesis awaits further research.

### 4.2. Indifference to Pain

Unlike wounded terrestrial mammals, injured dolphins are said to be indifferent to pain [1,60]. Faithful Canvas healing histories disconfirm this, based on several eyewitness accounts of newly injured dolphins striking the wounded body part or themselves against the water surface or writhing in pain around a conspecific ([12] Vol. 9 Peduncle Bites, Ch. 1, Valiant; Vol. 4 Dorsum Bites, Ch. 9, Rose). Similarly, an old, sickly dolphin severely lacerated by a propeller strike immediately showed significant agitation [10]. Dolphins react to the *approach* of a white shark with obvious agitation [62,201]. Whereas imputed pain indifference and any conceivable benefits from that state remain to be determined, there may be potential analgesic roles because nerves in dermal and upper blubber are not replaced until very late healing [6]. The absence of nerves could reduce pain awareness enough to allow a wounded dolphin to continue swimming and feeding, the latter essential because regular meals help the dolphin’s high metabolism stave off starvation [108]. Human scars also lack nerves [97]. However, replacement of the dolphin nerves in late healing is curious, given replacement of the epidermis and the dermal–epidermal junctions in early healing. Potential natural dolphin analgesics (isovaleric acid and organohalogens) remain to be explored.

### 4.3. Hemostasis and the Diving Reflex

Anyone familiar with how Klimley [202] and colleagues located white shark attacks on seals around the Farallon Islands of northern California—scanning the sea surface from cliffs for vast pools of blood, with a shark waiting nearby for its shocked prey to bleed out—must be struck by the lack of blood from fresh wounds on shark-bitten dolphins, much less bleeding out. None of the freshly wounded dolphins in the Faithful Canvas collection were seen bleeding [12]. In a controlled wounding study on bottlenose dolphins, blood coagulated visibly in superficial wounds within 2 h but not thereafter [15]. Few other studies of healing reported bleeding [9,10,78]. Given the highly vascularized collagen cargo of dolphin dermis and blubber, lack of bleeding implies notable clotting mechanisms.

The cetacean diving reflex may play a role in hemostasis [1]. Dolphin clotting is complex. Terrestrial animals respond to prolonged submersion with fatal intravascular clotting [203]. Cetaceans, however, regularly use prolonged submersion to feed. This requires their blood to flow in a way that accommodates the opposing needs of not clotting while submerged but clotting when wounded [51]. The diving reflex maintains blood flow while a dolphin is submerged [203] with a dramatic redistribution of cardiac output that reserves vital bloodborne oxygen for the oxygen-dependent brain and heart [49,50]. Specifically, blood pools in the unusually wide visceral venous channels and the complex vascular organ, the retia mirabilis; blood flow to body tissues such as lungs, muscles, and abdominal viscera is severely reduced; and non-essential blood vessels on the body periphery are narrowed (peripheral vasoconstriction). The breath is held (apnea). The heart rate slows (bradycardia, as low as 12 beats/min [51]). The magnitude of these routine cardiovascular adjustments during cetacean dives is only approached in terrestrial animals under pathological conditions, such as hypovolemic shock [204]. This key cetacean adaptation is an amalgam of independent adaptations [203]. In most mammals, Hageman factor (Factor XII) is a protein crucial for initiating blood clotting [52]. Dolphins lack Factor XII [46,51]. This key cetacean adaptation prevents blood clotting during dives but also markedly prolongs coagulation time in cetaceans compared to humans [51]. However, despite slow coagulation, if diving reflex mechanisms of central blood pooling, bradycardia, and peripheral vasoconstriction were triggered by wounding, they could conceivably limit blood flow to a wounded area and perhaps prevent blood loss from open wounds. Questions include the extent of cardiovascular adjustments among coastal cetaceans on short dives in shallow water.

### 4.4. Faithful Canvas Macroscopic Markers Reflect Immune Phases

Faithful Canvas pigment patterns and photos (Figure 3, Figure 4, Figure 5, Figure 6, Figure 7, Figure 8, Figure 9, Figure 10, Figure 11, Figure 12, Figure 13, Figure 14, Figure 15, Figure 16, Figure 17, Figure 18, Figure 19, Figure 20, Figure 21, Figure 22, Figure 23, Figure 24, Figure 25, Figure 26, Figure 27, Figure 28, Figure 29, Figure 30, Figure 31, Figure 32, Figure 33, Figure 34, Figure 35, Figure 36, Figure 37, Figure 38, Figure 39, Figure 40 and Figure 41) match and confirm published reports of healing stages (Table 2) and expand on Su et al.’s five stages [6,22,23] by providing estimates of each stage’s duration and illustrating their dynamic macroscopic changes in live recuperating dolphins. Matches make it plausible that the initial dark-to-light pigment pattern [12] is a macroscopic marker of overlapping inflammation and proliferation, its white borders serve as soft scabs that reflect ongoing inflammation, and the light-to-dark pigment pattern is a macroscopic marker of late proliferation and remodeling. Further, the evidence suggests that inflammation is prolonged, proliferation is accelerated, prolonged inflammation results in atrophic scars, and remodeling is the longest stage.

#### 4.4.1. Dark-to-Light Pigment Pattern Linked to Early Inflammation and Proliferation

Figure 3A–C, Figure 8A–C, Figure 15A, Figure 20A, Figure 22A, Figure 27A,B and Figure 37 which are from the Faithful Canvas collection, are fresh wounds in Su et al.’s stage 1 [6]. Starting some 6 h after wounding, fresh wounds are infiltrated by inflammatory neutrophils, which undergo apoptosis 6–12 h later and accumulate with dead and dying cellular debris, adipocytes, and collagen [15]. This creates a kind of conveyor belt of cellular carnage as long as microbial invasion continues. Some 1–3 days after wounding, the dark-to-light pigment pattern (the first) develops. Some 2–4 days after wounding, macrophages begin migrating to the wound to phagocytize dead neutrophils and microbes, stimulate continuing inflammation, and ultimately end their destructive cellular activity in favor of the constructive consequences of proliferation. The dark-to-light pigment pattern develops about the same time macrophages migrate to the wound site. Surgical wounds in the dark-to-light pigment pattern match Su et al.’s stage 2 healing wounds without visible granulation (Figure 4A, Figure 9A,D, Figure 10A, Figure 16A,B, Figure 17, Figure 21B and Figure 38). Gnarly wounds in the tri-colored dark-to-light pigment pattern match Su et al.’s stage 3 healing wounds with visible granulation (Figure 4B, Figure 9B,C, Figure 10B,C, Figure 21A,C,D, Figure 22D, Figure 28A,B, Figure 31, Figure 32 and Figure 39).

The conclusion that dark-to-light pigment patterns reflect overlapping inflammation and proliferation is corroborated by the parallel macroscopic and histological evidence of Bruce-Allen and Geraci [15]. It is further corroborated by macroscopic evidence in the three Faithful Canvas healing histories of chronic wounds, which exhibited dark-to-light pigment patterns during chronic wounding (Figure 4) and did not shift to the light-to-dark pigment pattern until healing began (Figure 5) ([12] Vol. 5 Dorsal Fin Slices and Scrapes, Chs. 2 & 3 Keynote Cases, Vidalia & Juno; Vol. 3 Head Bites, Ch. 2 Keynote Case, P).

Matches also make it plausible to suggest that the inner white border of the first dark-to-light pigment pattern functions as the soft protective scab first reported in 1985 [15]. It is plausible that it is co-opted from the necrotic cellular carnage of the original wound and maintained by a conveyor belt of cellular carnage while the wound is open. It is plausible that the inner white border is a macroscopic marker of overlapping inflammation and proliferation at sea. Sometimes called ‘slough’ [184], I use the term ‘white border’ instead of ‘slough’ to avoid confusion with the naturally high rates of skin sloughing in bottlenose dolphins. Su et al. [6] also used the term “white borders” (p. 13). Reports of blubber covering a wound (by the second day [1]; within a week [21]) may have been referring to white border soft scabs (Figure 31 and Figure 32).

Histologically, cellular carnage consists of dead and dying endothelial cells, inflammatory debris, adipocytes, and collagen [6,15,22,23,97]. In terrestrial mammals, cellular carnage dries and forms a hard scab that protects the fresh skin being formed beneath it. When fresh skin is ready, it dislodges the scab’s holdfast, and the scab falls off. The absence of hard scabs in dolphins implicates distinct inflammatory mechanisms, possibly resembling scarless fetal wound healing or genetic losses that altered conventional inflammation and proliferation dynamics, placing cetaceans as valuable models of wound healing and inflammation [44].

Accumulating white border soft scabs ostensibly protect underlying healing processes like natural bandages. In humans, closure of deep open wounds is hastened by skin grafts and occlusive dressings [185]. Occlusive dressings are waxy, nonabsorptive dressings that create a local environment that facilitates more rapid migration of epidermal cells to the wound for reepithelialization [205] and seal wounds and surrounding tissues from air, fluids, and harmful microbes such as viruses and bacteria [206]. In captive dolphins, wounds are protected with polyvinyl, hydrocolloid, and absorptive dressings [207]. It remains to be determined whether white border soft scabs serve as natural occlusive dressings and to what extent they protect against microbial invasion.

Among Faithful Canvas dolphins, white border soft scabs generally last 4–8 weeks (lasting 2.8 months in the most severe wound, Figure 37, Figure 38, Figure 39 and Figure 40) as they slowly shift into the second light-to-dark pigment pattern. Dolphin soft scabs are composed of necrotic tissue. Human and lab animal wounds with necrotic tissue do not heal [97]. This is why medical methods to remove necrotic tissues and bacterial bioburden are immediately mobilized to allow healthy tissue to grow [175], including antiseptics, antibiotics, and several debridement methods [4]. Natural removal includes inflammatory phagocytes that clean the wound and, in aquatic environments, constant irrigation (hydrodebridement). Thus, to last for weeks, the soft scab of degenerating cells must be balanced with conventional mammalian needs to debride necrotic tissue [97] and dolphin sloughing rates that are 8–9 times faster than human epidermal sloughing [19]. There may be a clue in the four layers of granulation [184]. The bottom fibroblast layer (50%) is overlaid by a capillary layer of inflammatory cells (25%), topped with loose avascular connective tissue (15%), and capped by slough (10%). The capillary layer may serve as an important buffer separating underlying fibroblast-collagen proliferation from the upper necrotic layer of inflammation. Notably, exuberant granulation does not show a layered organization [184], and I did not find any published information that free-ranging dolphin granulation is layered. How the loose cellular carnage of white border soft scabs stays glued also remains to be determined. However, the natural cellular adhesives of fibrin and fibronectin in the blood clot, the provisional matrix, and the new ECM in granulating tissue [92,97] could be leveraged to maintain the integrity of the cells in soft scabs.

Compared to humans, dolphin skin cells are produced at least twice as fast [44] and are sloughed 12 times faster [45]. Constant sloughing maintains a smooth surface as a front line limiting microbial colonization or biofouling [44]. Historically, the question of whether cetaceans’ skin possesses natural antimicrobial coatings was ignored because it lacks terrestrial mammals’ sweat and sebaceous glands, which actively participate in innate immune defenses by providing antimicrobial peptides, lipids, and other immunoregulatory barriers [19,157]. The propensity for skin infections that dolphins housed in chlorine- and ozone-treated pools develop suggests an imbalance between resident microflora, pathogens, and antimicrobial substances on the skin [19,208]. Cetacean skin microbiota may provide specific health- and immune-related functions across diverse ocean ecosystems [101,121]; e.g., the biogel on pilot whales (*Globicephala melas*) smooths the skin surface, enhances self-cleaning, and prevents organisms from settling [106]. However, more data are needed, and the natural mechanisms of dolphin debridement and sloughing must be reconciled with the durations of white-border soft scabs.

Some white border soft scabs change color from pale gray to bright white (the “brightening”), back to dull white, and eventually to the pale gray of the second pigment pattern (Figure 15, Figure 16, Figure 17 and Figure 18). The function of such brightening is unknown. However, it may signal the launch of the adaptive inflammation phase, whose highly discriminating capabilities improve via immunological memory [118,169]; it may result from influxes of unpigmented neoepithelialization from exponential mitotic increases in the dermal–epidermal junction [15]; or it may result from both, or neither.

Finally, in the initial dark-to-light pigment pattern, the timing of its outer dark border matches Su et al.’s [6,22,23] histology of initial hemostatic vasoconstriction and pooled blood, and subsequent proliferative angiogenesis. Thicker dark borders are potentially related to greater initial damage. However, dark borders also appear to be confounded with blubber thickness [12]. Blubber is thickest on the back, thinner on the sides, thinnest on the distal portions (head, peduncle), and either absent [128] or very thin [112] on the sides of the fins (fin faces) but thicker on their leading edges. Correspondingly, dark borders are usually fainter on dorsal fin faces (Figure 31) and heads (Figure 38), where blubber is thin, but are more vibrant on wounds on the body proper (Figure 9 and Figure 28) and leading edges of fins (Figure 4) where blubber is thicker [12]. The association between outer dark borders and blubber thickness remains to be determined.

#### 4.4.2. Light-to-Dark Pigment Pattern Linked to Late Proliferation and Remodeling

The light-to-dark pigment pattern is the second pigment pattern to develop. Matches between the Faithful Canvas [12] and Su et al. [6] suggest that the light-to-dark pigment pattern is a macroscopic marker of late proliferation and remodeling. By 4–8 weeks of healing, most Faithful Canvas wounds had preliminary seals (were new scars) and had switched to the light–dark pigment pattern with incomplete repigmentation. It takes 3–10 weeks for new skin cells to ascend from the dermal–epidermal junction to the surface [103]; the timing of the shift to the light-to-dark pigment pattern may be related. Figure 6, Figure 7A, Figure 11A, Figure 12A, Figure 13A,B, Figure 14A,B, Figure 18, Figure 19A,B, Figure 22B,C, Figure 23A–D, Figure 24A, Figure 25A,B, Figure 26, Figure 29A,B, Figure 30A, Figure 33, Figure 34, Figure 40 and Figure 41) match Su et al.’s descriptions of stage 4 new scars as closed wounds with contraction lines and incomplete repigmentation. Su et al.’s stage 5 scars are characterized as closed wounds, no contraction lines, full repigmentation to the background, and full cellular restoration. Stage 5 wounds had the fewest matches with Faithful Canvas [12] scars because many scars remain hyperpigmented for extended periods, and most are permanently atrophic, which do not meet Su et al.’s stage 5 criteria. Only 1 of the 106 Faithful Canvas healing histories tentatively meets Su et al.’s stage 5 criteria (Figure 35 and Figure 36). Both Su et al.’s stages 4 and 5 scars are characterized by gradual restoration of normal skin architecture, indicative of early and later remodeling, respectively.

Most Faithful Canvas healing histories suggest that bottlenose dolphin remodeling is the longest stage [12]. The light-to-dark pigment pattern fades very slowly, sometimes lasting for years. Enduring atrophism and evidence of scar aging are other examples. Given that cetacean swimming and diving could be compromised if cetacean wounds did not fill in like normal skin [6], especially for heavily scarred individuals (Figure 7, [12] Vol. 2 Body Bites, Chs. 10 & 11 Flash & Bugsy), an outstanding question is why so many Faithful Canvas dolphins have so many atrophic scars.

### 4.5. Extended Inflammation and Atrophic Scarring

A striking finding is that most of the Faithful Canvas scars [12] are conspicuously sunken, concave, or atrophic (Figure 6, Figure 7, Figure 13, Figure 14, Figure 23 and Figure 30). To the best of my knowledge, free-ranging bottlenose dolphins do not develop fortuna or keloidal scars. However, they develop one type of convex hypertrophic scar in the form of very rare comb tooth rake scars on dorsal fins ([12] Vol. 3 Head Bites, Ch. 9 Keynote Case, Fishlips). The harsh cosmetic reality is that the majority of Faithful Canvas bottlenose dolphin wounds heal into sunken, atrophic scars regardless of wound severity, which even become accentuated over time as scars stretch by widening [12]. Excessive scarring results from bacterial infection [6]. Thus, the central factor in dolphin healing is how inflammation prevents infection amid continuous microbial invasion of open wounds. Until sealed, open wounds on dolphins at sea are guaranteed antimicrobial depots. A persuasive example is the brutal shark bite that degloved an adult dolphin’s head so extensively (Figure 37, Figure 38, Figure 39 and Figure 40) that she survived against all odds; it took 2.8 months for her brutal bite to seal. Because the length of time a wound without medical intervention is open determines the duration of its inflammatory response [97], a plausible explanation for conspicuous dolphin scarring is that, at sea, dolphin inflammation continues to fight infection until the wound is sealed, and extended inflammation results in atrophic scarring.

The proposed extended inflammation in bottlenose dolphins is plausible for several reasons. One reason is its merit. Inflammation functions to clear wounds of microbes. It is unnecessary in sterile conditions [161,166,167]. All wounds are microbially colonized to some degree [2], although intentional surgical wounds in medicine and research are generated in near-sterile conditions. Unintentional traumatic wounds, whether predatory bites on extant free-ranging dolphins (Figure 37, Figure 38, Figure 39 and Figure 40) or ancestral humans, guaranteed microbial contamination until the wound was sealed (Figure 37). Szpaderska and DiPietro [166] speculated that open wounds required intense inflammatory activity that kept the wound open until inflammatory cells cleared the invading microbes, which prompted the evolution of wound healing to select the most beneficial response to microbial contamination: surviving with scars versus dying by sepsis. Thus, exuberant inflammation may be an advantage in open, microbially contaminated wounds versus a liability [166]. Unfortunately, in contemporary human contexts of sterile surgical wounds, exuberant inflammation with its harsh cosmetic outcomes [2] is often at odds with rapid scar-free repair [161].

A second reason why the proposed extended inflammation is plausible is the comparison with lab studies. In terrestrial mammals, hard scabs protect open wounds from microbial contamination. Using white border soft scabs as proxies for inflammation, Faithful Canvas [12] dolphin inflammation lasts 1–2.8 months. These estimates are longer compared to general estimates of inflammation in experimental healing studies (4–6 days [92]; 1–10 days [97]; 1–14 days [14]). Note that Table 1 lists estimated healing times, not inflammation. All that is known about inflammation in free-ranging dolphins comes from two studies. Unfortunately, Su et al. [6,22,23] could not estimate the duration of inflammation in stranded dolphins, and Bruce-Allen and Geraci [15] studied superficial wounds whose inflammation lasted only a few days. Durations of bottlenose dolphin inflammation are also expected to vary by time of year, dolphin age and health, water pollution, wound severity, body location, and differences in blood supply to the wound site (which in turn are affected by dehydration and seasonality).

Third, inflammation times vary even within related species, demonstrating the biological flexibility of the immune machinery across species. In beluga whales, *Delphinapterus leucas*, wound repair takes five times longer than in bottlenose dolphins [209]. In carnivores, granulation formation and wound contraction are slower in cats than in dogs [210]. In equids, pony inflammation is strong but short, leading to better-quality second-intention healing. In contrast, in horses, inflammation is weak and chronic, leading to the formation of poorly vascularized, exuberant granulation tissue and scarring [165]. Finally, inflammatory neutrophil counts can be uncorrelated with wound age, at times persisting well into proliferation [184].

In humans, atrophic scars represent some of the most difficult and insidious pathologies confronting the reconstructive surgeon [211]. Histologically, there is conspicuous forfeiture of collagen, elastin, and ECM with thinner dermis and epidermis after healing [16]. Pathophysiologically, collagen deficiency in atrophic scars is often due to impaired fibroblast function (which, in turn, impairs collagen production) and to enhanced ECM degradation by MMPs [16].

Atrophic scarring is directly related to the duration of inflammation [14,16,212,213]. In humans [2] and pigs [214], healing is delayed by sustained high bacterial loads, a sustained influx of proinflammatory cells, and the destructive consequences of robust acute inflammation [166]. Chronic human wounds result from prolonged, dysregulated inflammation driven by the continual presence of inflammatory leukocytes, primarily neutrophils. Excessive neutrophils impede repair by producing caustic enzymes normally targeted for microbial destruction that can also cause substantial collateral damage when directed at new tissue [166].

Why the majority of the Faithful Canvas [12] dolphin scars are conspicuously and permanently atrophic is unknown (Figure 7, Figure 11 and Figure 30). However, human atrophic scars develop through several processes [16], which support my proposed connection between prolonged inflammation and atrophic scarring. One, atrophic scarring is often triggered by an intense or prolonged inflammatory response, which in humans includes severe acne or chickenpox, that damages dermal collagen and elastin fibers. Two, the normal healing process in which fibroblasts produce and remodel new collagen is disrupted, so not enough new connective tissue is created to fully reinstate lost tissue. Three, the net loss of dermal volume is produced by an imbalance between new tissue synthesis and damaged tissue removal. Matrix metalloproteinases (MMPs expressed by macrophages, fibroblasts, and keratinocytes) digest collagen and destroy preexisting ECM in the wound area [92]. Unchecked, this enzymatic breakdown of ECM can overpower the body’s ability to create new tissue [16]. Nearby unwounded tissue is protected from enzymatic dissolution by protease inhibitors; however, inhibitors can also be overwhelmed by massive protease releases during extremely robust inflammation [92]. Four, the atrophic scar’s sunken appearance is due to insufficient collagen production and less supportive tissue beneath the skin, causing the skin’s surface to sink.

In contrast, low levels of inflammatory cells result in scarless healing in fetuses [161,188,215], mouth wounds [166], and genetically altered mice incapable of inflammation [166]. Although mechanisms of fetal wound regeneration remain unclear [161,167,168,216,217], several differences between fetal and adult wound healing ([161], Table 1 & 2) are potentially relevant to dolphin atrophic scarring. Compared to adult wound healing, fetal scarless healing has a reduced inflammatory response, but fetuses artificially stimulated to produce adult-like inflammation grow adult-like scars. Fetal wound ECM shows increased hyaluronic acid levels for longer durations. There are increased levels of MMPs and reduced levels of their inhibitors. Increased collagen III expression restores the normal basketweave pattern of uninjured skin. In fetal scarless healing, TGFβ levels are lower and cleared more quickly, PDGF levels are elevated and cleared more quickly, and epidermal growth factor (EGF) induces mitosis in several cell types, including fibroblasts and keratinocytes, two major cell types in granulating tissue [97]. The role of angiogenesis and VEGF remains unclear [161]. Fetal scar-free healing is associated with elevated levels of PRP and lower levels of VEGF and TFGβ [164]. Compared to human adults, dolphins have comparable PRP levels but significantly lower levels of VEGF and TFGβ [46]. This introduces apparent contradictions. One, dolphins have reduced levels of proliferative TFGβ, which, in other models, reduces scarring, yet dolphins scar conspicuously. Two, fetal wounds show increased levels of MMPs with reduced levels of their inhibitors, a cellular situation that results in matrix degradation, accounting for atrophic scars in adults [16], yet fetuses heal scar-free [161]. Three, dolphin skin and dermal–epidermal junctions are replaced early in healing [15], yet underlying tissue is not replaced [12], despite dolphins’ accelerated production of skin and adipose stem cells.

Given the extensive matches and confirmation between Su et al.’s [6] healing stages and the Faithful Canvas collection [12], the mismatch with the literature on atrophic scars is striking. Few publications mention atrophic scars, whereas the Faithful Canvas collection is replete with them. Bloom and Jager ([10] Photo 7), described propeller strike scars as recessed areas with pale curved scar lines surrounded by much darker pigmentation, described in Faithful Canvas terms as atrophic scars in the light-to-dark pigment pattern with hypopigmented riverine scars. Corkeron et al. characterized shark bite scars on dolphins as extensive deformations [9,60].

If dolphin inflammation is prolonged to cope with microbial invasion, as long as wounds remain open, their inflammatory stop signals may differ from those in terrestrial models. A potential puzzle piece is that dolphin inflammation is spatially constrained histologically [15]. Su et al. [6] reported a tiny 4–7 mm zone of mitosis and inflammation around stage 2 wounds, arguing that pronounced mitosis of the *Tursiops* dermal–epidermal junction apparently provides enough germinal cells in the immediate vicinity of a wound to fill its gap from a very short distance. Though reminiscent of the convergence of granulation islands emanating from hair follicles to fill the wound [183], atrophism shows that the wound lumen is filled with scar tissue incompletely.

More data are needed on the durations of dolphin inflammation, if/how extended inflammation maps onto atrophic dolphin scars, and key events that signal inflammatory waning, inhibit neutrophil infiltration, and promote macrophage polarization to an anti-inflammatory phenotype [92].

### 4.6. Blubber and Stem Cells

Cetacean blubber is a unique, thick, thermoregulatory cushion that streamlines the body, nourishes it during food scarcity, and protects its internal organs [47,127,132]. The dolphin blubber layer is many times thicker than the human subcutaneous adipose layer (Figure 1) and chemically stratified (e.g., collagen [23]; isovaleric acid [131]). In healing, blubber plays a mechanical role by shielding the underlying musculature from damage. It may serve a hemostatic function when it protrudes from cutaneous wounds (Figure 8). However, despite several studies on its function, development, and morphology [47,127,129], a detailed study of wounded blubber has not been undertaken. Its potential biochemical roles in wound healing are unknown. However, several blubber features could contribute to variation in healing rates. One, blubber thickness varies with body location [126]; *Ceteris paribus*, of two identical 25 mm deep wounds from a bull shark tooth, the one penetrating thicker blubber does less damage. Two, blubber chemistry changes across seasons and lifespans [133,134]. Three, there are sex differences in blubber thickness [134,135]. Four, the blubber’s dense vascularization and specialized stents for enhanced flow [47,124] may expedite the flow of inflammatory traffic to the wound, although blubber itself blocks cellular traffic [97]. Finally, blubber is a source of adipose stem cells, whose plasticity could contribute greatly to healing.

The enticing potential of stem cell plasticity in healing remains elusive. Current knowledge is mainly derived from murine models, including genetically modified mice amenable to lineage tracing; the extent to which murine model lessons apply to humans (or dolphins) remains to be determined [97]. Possible cellular sources of post-wounding adipocytes remain highly debated [218]. Finally, rather than a systematic identification of all cell types adipocytes can transdifferentiate into, current knowledge of adipocyte plasticity is based on isolated findings across different disease models [218].

Progenitor bone marrow stem cell populations give rise to major leukocyte subpopulations (neutrophils, eosinophils, monocytes and macrophages, lymphocytes [118]). Alternatively, stem cells can accelerate healing by modulating the immune system [219], potentially reducing inflammation by secreting anti-inflammatory factors such as TGFβ, inhibiting lymphocyte proliferation, inducing macrophages in vitro, and generating immunoregulatory types of macrophages in vivo [152].

What little is known about adipose stem cell reprogramming also emanates from murine models. Adipocyte regeneration is yet to be fully elucidated, but most adipocytes can undergo cellular reprogramming [218]. Interconversion of adipocytes and endothelial cells is a possibility [220,221]. Mouse adipocytes exhibit cellular reprogramming into preadipocytes and mesenchymal stem cells by dedifferentiation (a differentiated cell reverts to a cell with a greater developmental potential, such as into a stem or progenitor cell), or into myofibroblasts by transdifferentiation (a differentiated cell transfers to another type of differentiated cell [218]). The human skin stem cells that ensure continual epidermal turnover, which maintains skin homeostasis and healing, are in the bulge of pilosebaceous units (hair follicles and sebaceous glands) and interfollicular epidermis [104]. Follicular stem cells can differentiate into sebaceous glands or interfollicular cells [97]. In dolphins, the evolutionary loss of hair follicles and sebaceous glands means that their roles in terrestrial mammal wound healing are replaced by alternative mechanisms in dolphin healing.

Adipocyte stem cells mediate fibroblast function, which is crucial for wound repair because they produce collagen [222], but questions remain about whether adipose tissue around full-thickness wounds supplies essential cells to granulation tissue [6]. In Su et al.’s [6] stage 2 and 3 wound characterizations, blubber was replaced by a loose fiber network of fibrin, erythrocytes, and inflammatory cells. Adipocyte disappearance during wound healing in cetaceans might result from apoptosis, dedifferentiation, or transdifferentiation [218]. If adipose stem cells in cetacean skin are essential sources of granulation tissue, adipose regeneration would be necessary to help heal future wounds [6]. In Su et al.’s [6] stage 4 and 5 wound characterizations, blood vessels were surrounded by small, rounded, membranous spaces. Su and colleagues hypothesized that these could be regenerating neo-adipocytes. However, given extensive Faithful Canvas [12] matches suggesting that stage 4 and 5 scars were months to years old, such adipocyte replacement would be very late in the remodeling process. It remains to be established whether adipocyte stem cells are exhausted without replacement during the healing process [46]. Atrophism suggests that they are spent without replacement [16].

Further work is needed to understand the role of stem cells in fetal and adult wound healing [23,161], the sources of fibroblasts in the absence of wound fibroblasts [97], how tissue forfeiture relates to cetacean inflammation [16], and the mechanisms by which cetacean wounds remain atrophic.

### 4.7. Hyperpigmentation, Melanocytes, UV Protection, and Immunity

Once new scars develop, the second light-to-dark pigment pattern develops after some 4–8 weeks of healing; hyperpigmentation on some scars lingers for months or years before repigmenting to the background color (Figure 6, Figure 14, Figure 24, Figure 29, Figure 36 and Figure 41). The longest to linger, 14 years, was from a jagged shark bite on the head of a neonatal dolphin (Figure 6). Another jagged peduncle bite on a calf took 8 years to match the background [12]. Years of repigmentation to match the scarred dolphin’s background color are the last visible part of the final remodeling, suggesting that remodeling is the longest dolphin healing phase, that repigmentation was the last priority in the evolution of odontocete healing at sea, and/or that long-term hyperpigmentation provides benefits beyond UV protection [12]. Whatever their ultimate roles, melanocyte and melanin replacement in early healing attests to their importance [6,15,22]. Faithful Canvas [12] evidence suggests that enduring scar hyperpigmentation in dolphins serves dual purposes: protection against UV radiation and microbial invasion, and defense of weaker scar tissues as immune sentinels.

Faithful Canvas [12] healing histories confirm Su et al.’s [6,22,23] partial repigmentation of stage 4 scars and full repigmentation of stage 5 scars with the caveat “as long as enough time has passed.” Like atrophic scars, the mismatch with the literature on prolonged scar hyperpigmentation is striking. Few mentioned variation in pigmentation [20,22,60], and no publication to my knowledge mentions enduring scar hyperpigmentation.

Skin pigmentation is a sophisticated biological process involving multiple genes that can be modulated by several intrinsic and extrinsic factors [22]. Pigmentation stems from melanin, a pigmented biopolymer produced by organelles inside melanocytes called melanosomes via melanization; melanin accounts for hair and skin color [122]. Normal skin pigmentation also relies on several critical biological steps, including differentiation of melanoblasts into melanocytes, melanocyte migration from the dermis to the epidermis, and transfer of melanosomes from melanocytes to keratinocytes [213].

In humans, abnormally pigmented scars have long been clinical problems for clinicians and impose substantial economic and psychological problems on patients [211,223]. Abnormal pigmentation is classified as hypopigmented (lighter than background), hyperpigmented (darker than background), or mixed [224]. Hyperpigmented scars often result from the chronic wounds of systemic disease complications like diabetes, heart disease, and aging [213]. In humans, the underlying mechanisms of repigmentation remain largely unknown [22,213,225], making hyperpigmented scars difficult to treat.

For example, hyperpigmentation is mostly but not wholly a function of skin pigmentation. As expected, human populations with darker skin have higher melanin levels than Caucasians [186], and melanocyte density is greater in darker than lighter skin in Fraser’s dolphins [22]. In experimental Duroc pigs, scar pigmentation depends on wound width and depth; melanin takes twice as long to migrate into the scar centers of full-thickness wounds as in partial-thickness and incisional wounds [226]. Bottlenose dolphins have high levels of melanin, which accounts for their gray color [19] and should account for enduring hyperpigmented scars. Unexpectedly, many hypopigmented scars also develop [12]; they lack pigment despite rapid melanin replacement during early healing [19]. Risso’s dolphins (*Grampus griseus*) are also gray but notable for accumulating dense permanent coats of hypopigmented scars [137]. In Duroc pig scars, microscopic melanocyte deposition is *uncorrelated* with macroscopic appearance: After 90 days of full-thickness wound healing, the dermal–epidermal junction had a dense layer of melanin histologically, but the scar center was still hypopigmented macroscopically [213]; such scars are examples of the Faithful Canvas [12] dark-to-light pigment pattern. More bafflingly, in red Duroc pigs, there was no significant difference in the number of melanocytes between hyperpigmented and hypopigmented scars [213].

Melanin is a solar shield. When melanin absorbs UV radiation, increased melanocyte numbers and activity cause the skin to turn brown [113]. Melanin protects keratinocyte DNA from UV radiation [113]. Yet human scars must be protected from the sun for up to a year to avoid UV damage that can delay healing and create permanently thicker and more noticeably discolored scars; interestingly, hard scabs provide substantial solar protection [227]. In cetaceans, dark-colored whales show more signs of solar protection than light colored whales [123,228,229]. Presumably, new dolphin scar tissue also needs UV protection. Yet dolphin scars on body parts reliably above the waterline when their possessor surfaces to breathe are exposed to regular UV radiation. As solar shields, the flat faces of scar beds are expected to have darker pigmentation than the sides because they receive more direct sunlight, which is the Faithful Canvas bi-colored light-to-dark pigment pattern. However, tri-colored light-to-dark pigment patterns have hypopigmented interior riverine scars that take months or years to eventually fragment into variegated pigments (Figure 29) and ultimately develop enduring hyperpigmentation. Though at times shadows across atrophic tissues artificially accentuate hyperpigmentation, dark coloration compared to the background is visible in scars that stretch by widening. Scar hyperpigmentation that may have arisen from unavoidable solar exposure might also provide ongoing solar protection.

Yet UV exposure does not explain all hyperpigmented dolphin scars. In Fraser’s dolphins, melanocyte density was more related to skin pigmentation than anatomical location vis-à-vis UV exposure [22]. Scars that receive UV radiation every time the dolphin surfaces to breathe (i.e., dorsum from blowhole to and including dorsal fin) do not inevitably hyperpigment [12]. Some scars that should be hypopigmented develop dark hyperpigmentation instead. Female bottlenose dolphin Sahara was a yearling when her ventrum was sliced open by boat propeller blades (Figure 41). Dolphin ventrums are white due to countershading [39], though temporarily pink when socializing, as in Figure 41. Thus, Sahara’s ventral scars present a curiosity because they are not hypopigmented like her stomach; they are instead strikingly hyperpigmented. This contradicts Su et al.’s [22] finding in Fraser’s dolphins that white locations repigmented to white, whereas gray locations repigmented to gray. However, it aligns with their finding that melanocyte density was less closely related to anatomical location than to UV exposure. The dolphin ventrum is rarely/briefly exposed to the sun, so hyperpigmented ventral scars raise questions about whether UV protection is the sole role of hyperpigmentation.

Regardless of sun exposure, the repigmentation time needed for Faithful Canvas [12] hyperpigmented scars to eventually match the dolphin’s background color is highly variable. An apparent contradiction is that melanocyte populations in dolphin skin are restored very early in healing [15,19], confirmed 37 years later by the identification of melanocytes and melanin in migrating epithelial tongues during early Fraser’s dolphin healing [22]. In contrast, melanin does not occur in new pigmented epidermis from full-thickness wounds on lab animal models until scars are fully healed (reepithelialized [213]). Dolphins differ further in that their melanocytes lodge in the deepest epidermal layer, the stratum basale [230], and migrate to the outer stratum externum during healing [6], whereas human [97] and Duroc pig [213] melanocytes lodge in hair follicles and migrate into neoepidermis from them or wound edges.

Some variation in hyperpigmented Faithful Canvas scars is partially accounted for by wound type. Gouging (Figure 31) and degloving (Figure 23, Figure 24 and Figure 25) wounds that scoop or scrape off larger patches of skin surface heal to more hyperpigmented scars ([12] Vol. 6, Dorsal Fin Gouges; Vol. 9, Peduncle Bites). Deeper portions take longer to repigment than shallow portions (Figure 31 and Figure 34). Plus, hyperpigmentation appears to vary inversely with blubber thickness of the wounded area [12]. Specifically, wounds on body locations with thin blubber layers, such as the head, fins, and peduncle ridge, remain more conspicuously hyperpigmented longer than scars on the body proper.

Although melanin serves as a cetacean solar shield [123], the facts that some skin infections are more common on fair- versus dark-skinned people [231] and melanin is involved in invertebrate and teleost [232,233] immunity suggested that melanocytes and melanization have immune functions [231]. Accumulating evidence suggests that melanocytes and melanin have antimicrobial and immunomodulatory properties that actively participate in healing [113,122,234]. Zebrafish melanocytes stimulate inflammation during very early stages of healing [235,236,237]. In humans, there are several possible avenues for melanocytes to participate in cutaneous immunology [113,122]. Their dendritic nature, large surface areas, and strategic positioning in the epidermis with keratinocytes and Langerhans cells offer opportunities to encounter harmful microbes and usefully serve as early immune sentinels [113,122]. Normal human melanocytes express toll-like receptors (TLRs) that recognize pathogenic microbes and produce several proinflammatory cytokines and chemokines that modulate various skin immunity [238]. Melanocytes are capable of phagocytosis [113], a critical dimension of inflammation. Stimulated melanocytes secrete several proinflammatory cytokines and chemokines that affect both inflammation and proliferation, including keratinocytes, lymphocytes, fibroblasts, mast cells, and endothelial cells in the skin [239]. Melanin is a powerful antioxidant [213]. Melanization involves many toxic intermediates (e.g., the transfer of acidified melanosomes to keratinocytes) that are believed to exert strong antimicrobial effects [240].

More evidence of a connection between enduring dolphin hyperpigmentation and prolonged inflammation is that the inflammatory response is essential for wound hyperpigmentation [237], as hyperpigmentation in grafted skin accompanies a marked increase in levels of melanogenic enzymes and peptides [225]. However, this and other immune functions of melanocytes in cetacean skin warrant new studies [22]. Dolphin data are needed on factors that account for the considerable variability in the extent and duration of scar hyperpigmentation, the relationship between repigmentation rates and water temperatures, the distribution of hyperpigmented scars across the body and their relationship to solar protection, and the immunological roles of melanin. Cetaceans may present a novel approach to the study of wound healing and repigmentation in tight-skinned animals [6,22].

### 4.8. Challenges and Future Research

Efforts to monitor wounding and healing in free-ranging cetaceans are rife with challenges both fascinating and demanding. Healing studies at sea are hampered by no knowledge of wound age when first observed and sighting gaps where a wounded dolphin is not resighted for months or ever again [9,20,21]. Studies are also hampered by the most unsuspected Faithful Canvas finding, photographic laterality, a characteristic behavior in which a dolphin tends to show only one side of its body to the boat during a sighting. If the dolphin is wounded, its wounded side may or may not be visible to observers.

This study examined many chemical characteristics of cetacean skin and blubber, including multiple antimicrobials and stem cells, which call for future research on dolphin healing. Numerous other looming questions were unaddressed due to page limitations. One is the relationship between dolphin healing and the glucose, lipid, and amino acid metabolic reprogramming that fuels healing from inflammation to remodeling [182,241]. A second is the relationship between accelerated glucose production upon wounding to support cellular activity during healing [182,241] and dolphins’ ability to control insulin resistance [242]. Among humans, chronic [unhealing] wounds are most often associated with diabetes [2]. Dolphins can turn insulin resistance on and off without medical intervention, a practical adaptation for maintaining high glucose levels in the brain while living on a high-protein/low-carb diet that can involve long fasts between meals [242]. The connection between dolphins’ demonstrable success in healing without medical intervention, manipulatable insulin resistance, and the impacts of diabetes on chronic human wounds remains to be determined. A third is the relationship between vitamin A, its retinoids (e.g., retinol), which are crucial for the development of both innate and adaptive immune cells, and several fish species in dolphins’ piscivorous diet, which are good animal sources of Vitamin A. A fourth is the interactions of the pH of wounds, immune chemicals, unwounded skin, and seawater. A fifth is the fundamental requirements of delivering oxygen to the wound site and the role, if any, of the large oxygen stores in dolphin muscle myoglobin. Dolphin muscles contain many times more myoglobin than terrestrial animals [39], which hold large reserves of oxygen that are stored much longer than oxygen in blood hemoglobin [48]. A final example occurs among Indo-Pacific bottlenose dolphins (*T. aduncus*) that rub particular body parts against selected corals and sponges in the Egyptian Northern Red Sea, providing evidence of potential self-medication or auxiliary treatment against microbial infections [160].

## 5. Conclusions

Faithful Canvas [12] healing histories, scar searches, and standardized vocabulary are new tools for studying free-ranging bottlenose dolphin healing, natural history, and conservation with implications for human scarring. They reveal two pigment patterns visible to boat-based observers that indicate current immune stages of wounds on free-ranging dolphins. Further, Faithful Canvas data on wounds can inform veterinary pathology and forensic cases. Comparisons with captive dolphins can inform the influences of treated pool water, medical intervention, nutritious diets, and the absence of predatory pressures on healing. Comparisons with captive healing can also inform potential impacts of contemporary stressors on sea life on healing, immunosuppression, and increased susceptibility to infection. Stressors include excessive anthropogenic activities, shipping lanes, marinas, ports, coastal construction, chemical pollution, noise pollution, and heavy recreational and commercial boating [10,191,192,243,244,245,246]. There is much more to learn. As research continues and our understanding grows, current perceptions and priorities of importance will undoubtedly change.

## Data Availability

Data supporting my findings can be accessed in the ebook, Dolphin Healing at Sea without Medical Intervention: The Art and Science of Skin as a Faithful Canvas, when it is published in 2026.

## Abbreviations

The following abbreviations are used in this manuscript:

ASC	Adipose stem cell
ECM	Extracellular matrix
FGF	Fibroblast growth factor
IGF	Insulin-like growth factor
MMP	Matrix metalloproteinase
PDGF	Platelet-derived growth factor
PRP	Platelet-rich plasma
TGFβ	Transforming growth factor beta
TIMP	Tissue inhibitor of metalloproteinases
VEGF	Vascular endothelial growth factor
